# Comparing to a Neurotypical Norm Is Normal in Autistic Language and Communication Research: A Cross-Sectional Systematic Review and Critical Analysis of Recent Literature

**DOI:** 10.1177/23969415261441522

**Published:** 2026-05-24

**Authors:** Harrie Reynolds, Titia Benders, Josje Verhagen

**Affiliations:** 1 School for Cultural Analysis, University of Amsterdam, Amsterdam, The Netherlands; 2 Center for Language and Communication, University of Amsterdam, Amsterdam, The Netherlands

**Keywords:** Autism, neurodiversity, methodology, language and communication, comparative study design, systematic review

## Abstract

**Background and aims:**

Autism research has traditionally been shaped by the idea that autistic people have “deficits” compared to neurotypical people. This approach often involves directly comparing the two groups, with the expectation that autistic people will perform worse. However, neurodiversity advocates argue that autism should be seen as a valid way of being, and should not be defined in contrast to norm-centred ideals. This review aimed to assess to what extent autism research in the field of language and communication is structured around such norm comparisons.

**Methods:**

Following PRISMA protocols, a cross-sectional systematic review methodology was used to gather a sample of 249 relevant articles published in 2023. Articles were assessed as to whether they included a neurotypical comparison group and how the autistic group was expected to perform in contrast to neurotypicals. Additionally, we coded whether the study was an intervention, and whether non/minimally speaking (NMS) participants or those with intellectual disability (ID) were included, as we expected patterns to differ in these cases.

**Main contribution:**

We found that almost half (49%) of the studies compared autistic and neurotypical participants, and this rose to 75% when interventions and studies involving ID or NMS participants were excluded. When predictions were made about which group would perform better, 60% of studies expected autistic people to do worse, while only 4% expected them to do better. Intervention studies were less likely to make direct comparisons, and studies involving ID or NMS participants also tended not to use neurotypical comparison groups.

**Conclusions:**

Overall, our findings show that autism research is still largely built on comparisons with neurotypical norms, reflecting an assumption of deficit.

**Implications:**

For research to align with the neurodiversity perspective, future studies will need to move away from these deficit-based traditions. Suggestions for alternative, less norm-based approaches are discussed.

## Introduction

Neurodiversity is a growing area of interest within academia—a simple search for the term in the *Web of Science* database returns only one hit from 2004, but by 2024 this jumps to 441 hits. “Neurodiversity” refers to the idea that human neurology and cognition is a naturally varied system, analogous to biodiversity, and that there is no “correct” neurotype, although some dominant ways of functioning are typically favoured in our current society (Bertilsdotter Rosqvist et al., 2020; Chapman, 2023; Walker, 2021). The neurodiversity paradigm is often contrasted with the traditional—and still predominant—medical model (e.g., Chapman, 2020b; Walker, 2021). The medical model favours a norm-based approach and labels those outside of certain (revisable) boundaries to be “disordered,”^1^ and is therefore rooted in notions of pathology (i.e., DSM-5; American Psychiatric Association, 2013).

While neurodiversity is a wide umbrella term covering various ways of being, its conception can be traced back to the online autistic community of the 1990s (Botha et al., 2024). The neurodiversity movement began because many autistics felt side-lined and misunderstood by societal views of autism and the lack of autistic voices in research (e.g., Sinclair, 1993; Williams, 1996). Since then, the neurodiversity movement has grown and entered academic discourse, giving researchers a new paradigm to work with (Bertilsdotter Rosqvist et al., 2020; Walker, 2021). Advocates of this new paradigm claim that it not only offers a fairer and more humane view of autism and other neurological “conditions” than the medical model, but that it also promises new directions for research in various fields (Manalili et al., 2023; Pellicano & den Houting, 2022). For example, by taking the perspective that (mis)communication is bidirectional (following Milton, 2012), research can reconceptualise communication from an individual skill to an interactional phenomenon (Yu & Sterponi, 2023). Alternatively, new quantitative measures can be developed based on autistic experience (e.g., Conversation Questionnaire, Wilson, 2022).

Such new directions should ultimately be welcomed as, under the medical model, research has been prolific and yet is simultaneously floundering (Pellicano & den Houting, 2022; Rutter, 2014; Verhoeff, 2015; Waterhouse, 2013; Walsh et al., 2011). When established scientific “orthodoxy” is questioned, paradigms shift, and real scientific progress is made (Kuhn, 1962). It appears that autism research is on the cusp of such a moment (Pellicano & den Houting, 2022), and a neurodiversity paradigm may become the new norm. However, as Stenning and Bertilsdotter Rosqvist (2021, p. 1356) remark, “[w]e don’t yet know what epistemic or methodological rules will help us to get here.” In this paper, we aim to assess to what extent autism research in the field of language and communication is methodologically entrenched in the medical model through favouring negative comparative study designs, by which we mean designs where an autistic group is compared to a neurotypical “norm” to discover “deficits.” If a neurodiversity paradigm is to be embraced in research, this methodological practice must be challenged, and significant changes must be made to how empirical studies are conceptualised and designed. This paper aims to be a step in this direction.

“Autism Spectrum Disorder” has been medically defined by “deficits” in communication and social interaction, as well as restricted and repetitive behaviours and interests (American Psychiatric Association, 1985, 1998, 2013). Language and communication is therefore a core aspect of autism and an important topic for research. This research has typically focused on differences in specific language domains, such as pragmatics (Ying Sng et al., 2018), prosody (McCann & Peppé, 2003), and structural language (Boucher, 2012). Alongside attempts to identify impairments in these domains, research has attempted to link performance to wider cognitive “deficits,” such as problems with theory of mind or executive function (André & Maintenant, 2024), and to determine the relationship between autism and other neurodevelopmental conditions related to language, such as Specific Language Impairment (Félix et al., 2024).

Starting from the medical definition of autism, research has typically focused on detecting and further elucidating “deficits,” seeking their biological underpinnings and, eventually, “remediating” their effects through intervention (Hipólito et al., 2020; Verhoeff, 2015; Walsh et al., 2011). By following this line of enquiry, researchers frequently search for a core “deficit” that explains the “symptoms” of autism (Hipólito et al., 2020). After more than 80 years of searching, researchers have admitted that the heterogeneous presentation of autism is too diverse for any single aetiology to account for (Happé et al., 2006; Ure et al., 2018; Waterhouse, 2013). Language use across the spectrum exemplifies this heterogeneity, with some autistic individuals exhibiting fluent speech, while others experience language delays and/or challenges with structural language components and still others do not develop speech at all, or only minimally (see e.g., Vogindroukas et al., 2022 for an overview). Interestingly, this issue of heterogeneity and a missing aetiology is not only limited to autism, but almost all forms of “psychopathology” (Cramer et al., 2010; Harrington, 2019; Frances, 2009). This unyielding search for a cause of autism that cannot be found has been dubbed the “explanatory challenge” and has been likened to a “snark hunt”^2^ (Hipólito et al., 2020)—or in other words, a nonsensical quest. Although many researchers in the field have now accepted that universal deficits are a mirage, much research is still conducted as if such deficits existed and could ultimately still be found (Hens & Van Goidsenhoven, 2023; Stenning, 2023). Focusing research in this direction is arguably a waste of resources, but more importantly, it is creating a gap between academic pursuits and autistic lived experiences (Verhoeff, 2015).

In contrast, a neurodiversity-affirming communal definition of autism states that autistic traits are “natural variations of motivations and ways of being, that is, ways of perceiving, feeling, thinking, caring, moving, interacting, relating, and communicating” (Autistic Collaboration Trust, 2021). Differences in communication are therefore highlighted from this perspective too, but rather than focusing on autistic “deficits,” attention is often placed on the bidirectional nature of communication, known as the Double Empathy Problem (Milton, 2012). In this sense, it is not only the autistic who struggles to communicate with neurotypicals and who bears the responsibility of change, but also vice-versa. This view does not preclude helping autistic people improve their communication strategies. In an independent review of research priorities considered important by autistic people, their carers, and relevant clinicians, developing effective language and/or communication interventions were placed as a second priority, after mental health improvements (Cusack & Sterry, 2016).

Neurodiversity-affirming research begins with the assumption that “human neurological development is, at the species level, always diverse, unfinished and open-ended” (Chapman, 2023, p. 160)**.** Therefore, searching for one or more fixed “cause” would be contrary to the overarching philosophy. On top of this, autism—as a label or diagnosis—is recognised as a “moving target,” as its definition has changed over time and therefore cannot be dissociated from cultural values and social norms (Botha, 2025; Hacking, 2007; Stenning & Bertilsdotter Rosqvist, 2021). Thus, a neurodiversity-led view posits that autism cannot be regarded, as per the medical model, as a “monolithic natural kind” with an overfocus on discovering biological underpinnings (Stenning & Bertilsdotter Rosqvist, 2021). Rather, mixed- and multi-method interdisciplinary approaches are encouraged, recognising the interplay between various structures, including biology, culture, environment, dynamics, and discourse (Botha, 2025; Hens & Van Goidsenhoven, 2023).

Crucially, neurodiversity research rejects the practice of comparing autistic performance with an idealised “norm” to detect “deficits” (Chapman, 2023). This is in direct contradiction with the traditional medical model of autism, where a norm-based approach is typically applied, as to have a deficit, one must be deficient in comparison to some sort of “normal” level of functioning (Chapman, 2020a; Walker, 2021). Comparing functioning between individuals will always require (typically obscured) value-laden decisions about who to include in the reference class of “normal,” raising questions of objectivity and the risk of pathologising minorities (Chapman, 2020a). If, under a neurodiversity paradigm, the idea of a neurotypical “normal” is scrapped and research is no longer concerned with discovering so-called deficits in relation to this idealised notion, then comparative study designs will presumably become less prevalent in research. However, comparisons based within a positive framing of autism may still be relevant, for example when investigating (mis)communication as a bidirectional phenomenon rather than an autistic “fault” (Crompton et al., 2020; De Laet et al., 2025; Geelhand et al., 2025; Jameson & Bean, 2025; Milton, 2012). Alongside this, more research attention may be placed on the actual challenges faced by autistic individuals, with the intention of better understanding, and ultimately improving, lived experiences (see Ayaya, 2023).

A research programme that is centred around group comparisons additionally relies on assumptions of group coherence: that there is a shared factor that links individual members within a group and clearly separates them from non-members, therefore meaning that differences are generally smaller within groups than across them. These assumptions of coherence are essential for generalising from a sample to a presumed population and are not independently problematic, but may not mesh well with cases like autism where group coherence is conceptually questioned (Chapman, 2020b; Mottron & Bzdok, 2020; Timimi, 2016). The autistic group is vastly heterogeneous with no causal link or shared aetiology between members (Happé et al., 2006; Ure et al., 2018; Waterhouse, 2013), the definition of autism and its boundaries are shifting over time (Chapman, 2020b; Hacking, 2007), and observed differences between autistic and non-autistic samples have reduced accordingly (Mottron & Bzdok, 2020). Studies that attempt to learn about autism through group comparison will always be drawn into the murky waters of conceptualising what autism means on a group level. Even if we do assume that autism can define a group and that generalising from sample to population is therefore possible, this would require research samples to be representative of the whole autistic population and therefore free from sampling bias. Unfortunately, heterogeneity often gets in the way of this goal, as it can be difficult to create studies that include the full spectrum of autistic experience. This means that generalisations are potentially being drawn from incomplete data, an issue that has frequently been raised in relation to psychology research more broadly (Nielsen et al., 2017), as well as in the field of linguistics (Andringa & Godfroid, 2020). This point is particularly relevant for two autistic subpopulations: those with intellectual disability (ID), and those who are non- or minimally-speaking (NMS).^3^

Relatively recent estimates suggest that around a third (Shenouda et al., 2023) to a half (Katusic et al., 2021) of autistic individuals have an ID, a number that has decreased significantly over time (e.g., from 77.6% in 1996; see van Naarden Braun et al., 2015), in line with autism diagnoses becoming generally more prevalent (Fombonne, 2018; Zeidan et al., 2022). Despite the decrease in ID prevalence, ID remains far more present in the autistic than the general population, where estimates in the latter stand at less than 1.5% (Patrick et al., 2021). Estimates for the number of NMS autistics are somewhat more difficult to trace (Tager-Flusberg & Kasari, 2013), although one third is typically cited across the literature (e.g., Bryson, 1996; DiStefano et al., 2016; Jaswal et al., 2024; Koegel et al., 2020). This cited figure has remained oddly stable over the past thirty years, despite the broadening of the autistic definition, and evidence from population studies recording this statistic appears to be absent. Part of the problem in estimating the size of the NMS subpopulation is that there is little consensus as to what is considered to fall under this terminology, although suggestions towards standardisation have now been made, with recommendations for reporting standards and delineating terms according to age-normed language production percentiles (Koegel et al., 2020). More recently, NMS individuals and those with “severe” ID (IQ < 50) have been grouped together under a controversial (Kapp, 2023; Pukki et al., 2022) new term, “profound autism” (Lord et al., 2022). Prevalence of this subgroup has been estimated at around 25% (Hughes et al., 2023), further suggesting that one-third is an overestimation of the NMS population.

Regardless of the exact numbers and delineations, these subpopulations highlight the vast heterogeneity present within the autism spectrum and point to a variety of experiences and needs towards which research may be directed. Individuals with ID or who are NMS may experience significant and unique challenges that affect their quality of life (Gómez et al., 2020; Kaplan-Kahn et al., 2026), and therefore these challenges would benefit from separate research attention. At the same time, the design of studies and task demands often mean that NMS and individuals with ID cannot participate alongside their speaking peers or peers without ID.

Indeed, autism research has typically been skewed towards investigating participants without ID and with fluent speech. Specifically, Russell et al. (2019) found that across 301 research papers published in top autism journals in 2016, only 6% of autistic participants sampled had ID, and only 2% were NMS, suggesting a distinct underrepresentation of these groups. This underrepresentation matters, as Russell et al. (2019) point out, because the field is currently structured around the (idealised) assumption that knowledge gained from autistic samples can be generalised to the autistic population as a whole, and therefore samples should be free of selection bias. Thus, skewed samples create an unrealistic representation of the autistic population. Even if individual authors acknowledge the limitations of their specific samples, a cumulative research programme that consistently underrepresents areas of autistic experience will produce incomplete and skewed generalisations. When the results of individual studies cannot be reliably combined to inform us about autism as a group, it seems preferable to move away from group-based analyses. Instead, a neurodiversity-led approach that focuses attention elsewhere opens the way for taking diversity of experience seriously in research. Research in this vein could be concentrated on specific experiences, the results of which can be generalised to others with similar experiences, but not beyond this scope. In doing so, not only are problems of group generalisability side-stepped, but research is also then poised to include more diverse experiences, as constraints from norm-matching are lifted.

While the pathology paradigm seems to dominate academia (e.g., Chapman, 2020a), there is little quantification of this knowledge. In one recent systematic review, Bottini et al. (2023) analysed the use of terminology in 2322 articles on autism from 2021. Autistic advocates have argued that traditional medicalised language used to refer to autism (e.g., “Autism Spectrum Disorder”) is stigmatising, while identity-first language (e.g., “autistic person”) is neuro-affirming (Botha et al., 2023; Bottema-Beutel et al., 2021). Analysing the use of language to describe autism is therefore a window into how autism is conceptualised in research. In their sample, Bottini et al. (2023) found that traditional medical language was more common (70% of terms used across articles) than neuro-affirming language, but that this varied according to research topic, participant groups and journal specifics. These findings suggest that current research is still predominantly driven by a view of autism-as-pathology. However, while language use may be indicative of one or the other paradigm, it may also be, to some extent, superficial. For example, the authors found that articles published in journals with identity-first language guidelines naturally used more neuro-affirming language. Therefore, the use of these terms does not necessarily reflect how the authors themselves are conceptualising autism, especially as terminology may be tweaked as an afterthought. Other authors may repeat pathological terms simply because they are widespread in the literature. The use of terminology might not, therefore, be the best indicator of deeper systematic change or individual values. Furthermore, we can observe a cross-pollination of terms between paradigms without much meaning behind it. For example, it is common in autism research to label a comparison group “neurotypical”—the opposite of “neurodivergent,” and therefore a term that we would expect to be linked to neurodiversity-led thought—yet, the very notion of a comparison group is antithetical to the neurodiversity paradigm. It is therefore important to look beyond the words we use and consider methodological choices.

## The Present Study

As described above, the neurodiversity movement calls traditional methodological practices into question, especially those that revolve around detecting “deficits” in comparison to a neurotypical norm. However, we have little overview as to exactly how prevalent this style of research is. Given this, the present study aims to shed light on the prevalence of comparative study designs, specifically in the field of autistic language and communication. We consider this a relevant subfield as it concerns proponents of both paradigms; under a traditional medical model, autism is characterised by “persistent deficits in social communication and social interaction” (DSM-5; American Psychiatric Association, 2013, p. 50), while neurodiversity advocates may see autism as entailing “differences in subjectivity, communication, and thought” (Stenning, 2023, p. 6).

In a cross-sectional sample of empirical studies from this subfield published in 2023 and selected using systematic review methodology, we asked the following research questions: (a) do studies involve a direct comparison between autistic and neurotypical groups?, (b) in such comparisons, is the autistic group predicted to perform worse?, and (c) do practices differ in interventions and when ID and NMS subpopulations are included?

Considering that most research into autistic language and communication is presumably still couched in a pathology-driven medical model (Chapman, 2020a; Pellicano & den Houting, 2022), and assuming that norm comparisons are a core feature of this paradigm (Chapman, 2020a), we predicted that (a) the majority of studies would be designed around a comparison between autistic and neurotypical participants, (b) that most predictions would expect autistic subjects to perform worse, and (c) comparative study designs would be less prevalent in intervention studies as well as studies with ID and NMS subpopulations.

We expected intervention studies to typically omit comparisons between an autistic and control group because interventions often aim to “remediate” previously identified “deficits” within autistic participants. Thus, the focus is on increasing autistic performance to meet a previously established or assumed “normal” level. This might include standardised tests that are norm-scored, meaning norm-based comparisons are built into the study design without a direct comparison group being necessary. We analysed interventions separately within the sample to account for this potential difference.

We expected to find fewer comparisons between autistic and neurotypical groups in studies with ID or NMS subpopulations, without this being reflexive of either neurodiversity- or pathology-driven assumptions. In both these cases, the autistic subpopulation is already known to differ considerably from the “norm,” and therefore fewer comparisons with neurotypicals are expected. Additionally, research on these subpopulations may have a highly specific focus that is not relevant to neurotypical comparisons—for example, teaching Alternative and Augmented Communication (AAC) to NMS individuals. This would not only imply a research direction that is difficult to compare to “normal,” as most people do not communicate through AAC, but may also involve an intervention. Precisely because NMS individuals and those with ID are further from the “norm,” and therefore most in need of “correction,” intervention studies may be more prevalent with these subpopulations. Furthermore, NMS and ID are sometimes conflated into one group (i.e., “profound autism,” Lord et al., 2022), and therefore may be researched together. Therefore, we looked at these subpopulations in the sample to explore these nuances.

Although a systematic review methodology was employed to collect the sample of studies, following the Preferred Reporting Items for Systematic reviews and Meta-Analyses (PRISMA) guidelines (Page et al. 2021a, 2021b), the present study differs from a traditional systematic review (Grant & Booth, 2009) in two key ways. Firstly, rather than compiling an exhaustive history of all current knowledge in one (sub-)field, we analysed a snapshot of recent literature. Due to the breadth of the field and high number of relevant studies, we limited the search to one year of publication (2023) to ensure feasibility. This approach is similar to prior investigations into selection bias in research concerning clinical populations (Lee et al., 2007; Russell et al., 2019), and has been labelled a “cross-sectional review” (Russell et al., 2019). Secondly, the aim of the study was not to review the scope of current knowledge, but rather to critically evaluate methodological practices. Nevertheless, employing a systematic approach to collecting the sample ensured that the procedures were comprehensive, transparent, and replicable.

## Methodology

### Sampling

To detect potentially eligible studies, we compiled a list of keywords related to (a) autism; (b) language/communication; (c) empirical studies; and (d) human participants (see supplementary materials). We then identified 15 relevant articles published in 2023 in 15 separate journals and used these to check the coverage of our search terms in two large databases known to be appropriate for the field, *MEDLINE/PubMed and Web of Science.* Neither journal provided full coverage of the 15 articles alone but did so when combined. We therefore opted to use both. Search results from *Web of Science* (*n = *2026) and *MEDLINE/PubMed* (*n = *1586) were compiled and 1052 duplicates were removed using DedupEndNote (Lobbestael, 2023) and Zotero (2024) software. This resulted in an initial sample of 2560 articles. We restricted the search to articles published in 2023, but included articles that were available online from 2023 with later publication dates.

### Screening for Inclusion and Exclusion

The screening process had two stages, as is customary (Meline, 2006; Page et al. 2021a, 2021b). This involved an initial screening based on title and abstract, followed by full-text screening. To screen the titles and abstracts, we used ASReview (Van De Schoot et al., 2021), a software which uses an algorithm to reduce the number of articles that need to be manually checked. Through machine learning, ASReview sorts the uploaded articles by relevance after each manual decision is made. This means that studies marked as irrelevant by ASReview are placed at the bottom of the pile and may not need to be manually checked, saving valuable time. Studies assessing ASReview have found it to be both an efficient and effective tool (Chan et al., 2024; Van De Schoot et al., 2021).

Inclusion and exclusion criteria were based on four study aspects: (a) population; (b) use of primary empirical data; (c) outcome measures; and (d) research question(s)/aim(s). Between screening the abstracts/titles and full texts, the first and fourth criteria were revised and updated, as detailed below. These changes arose from reflecting on the process and identifying ways to better align the sample with our field of interest. Such a revision process is considered an acceptable practice in review methodology (Meline, 2006).

The first criterion focused on population, including autistic individuals with official, in-process, or self-diagnoses. In this definition, we aimed to be inclusive, recognising that obtaining a diagnosis can be challenging due to factors like gender biases (Haney, 2016) or ethnic minority underrepresentation (Begeer et al., 2009). Additionally, some individuals may be in the process of diagnosis, which can take years (Crane et al., 2016), and some may opt out of formal diagnosis (Kinderman et al., 2013). Studies involving other populations alongside autism (e.g., ADHD) or broader populations including autistic participants (e.g., ID) were included, provided results for autistic participants were distinguishable from the rest of the sample. After the first round of screening, we re-evaluated and updated our criteria to exclude infants “at risk” of autism, as this category is broad and autism may not be implicated. However, studies with “at-risk” infants who later received a diagnosis were included if other criteria were met. We also updated our criteria to exclude studies on autistic traits in the general population. Although participants with high traits could be considered equivalent to self-identifying or even diagnosed autistics, such studies did not necessarily divide participants into high- and low- trait groups. In these cases, the autistic (or autistic-adjacent) results were not distinguishable from the rest of the sample and thus did not meet this aspect of the criterion, as outlined above. We did not want to include only general population studies that did split participants into groups by trait level because this would mirror our dependent variable (inclusion of a comparison group). Consequently, we excluded all autistic trait studies conducted within a general population sample.

The second criterion—use of primary data—was met if the study reported empirical data obtained by the researchers. Therefore, any reviews, meta-analyses, and theoretical papers were excluded.

The third criterion concerned the study outcome measures. For a study to be considered relevant, it had to measure or describe autistic language/communication, as this was our subfield of interest. This definition included both qualitative and quantitative approaches, from single case studies to group analyses. Autistic language/communication could be measured directly (e.g., through direct tests or speech recordings) as well as via another's perception (e.g., parent questionnaires reporting perceived language/communication abilities). Studies that focused on non-reciprocal communication of a non-autistic person towards an autistic individual (e.g., parental speech) were outside of the scope, as they did not include samples of autistic language or communication.

The fourth criterion assessed whether at least one research question or aim of the study focused primarily on language/communication. This was only applied during the full-text screening stage, because we needed access to the full text to assess this aspect. Explicit aims and research questions were identified, and if unclear, independent and dependent variables were considered. To be included, a study needed at least one aim or question explicitly investigating autistic language/communication. Studies measuring the effect of non-linguistic variables (e.g., sleep) on general language/communication ability, or vice versa, were excluded for being too broad. Studies investigating non-linguistic variables through linguistic/communicative stimuli manipulation (e.g., verbal/non-verbal instructions on IQ scores) were included. We distinguished between studies measuring autistic language (e.g., linguistic abilities or development, communication style) and those including language/communication as a subcomponent of broader measures (e.g., symptom severity). The former were included for their specific focus on autistic language/communication, while the latter were excluded for their broader scope and limited information on language/communication—often only reporting group means on standardised (sub-)scales.

Finally, articles not in English were additionally removed, as were articles published in 2022 that were presumably retrieved due to a metadata error. We included studies that were published in any journal and did not include theses or book chapters.

During screening, the articles were blindly double-coded by two reviewers in iterative processes until interrater reliability was deemed sufficiently high (Cohen's kappa > 0.8, Cohen, 1960; see also Hallgren, 2012). At the first stage of title and abstract screening, two reviewers (HR & JV) coded samples of 100 articles as relevant or irrelevant using Rayann software (Ouzzani et al., 2016). After the first round, agreement was at 92%, and kappa was calculated as 0.67. The criteria were discussed, clarified and adapted between the reviewers and a second round was conducted. In the second round, 95% agreement was reached, with kappa equalling 0.87, completing the iterative process.

Once interrater reliability was achieved at the title/abstract level, all 2560 articles (including the 200 from the interrater reliability phase) were uploaded to ASReview software (Van De Schoot et al., 2021). Prior knowledge was uploaded to train the initial active learning cycle, in the form of five relevant and five irrelevant articles manually coded, and the default active learning model was selected due to its consistently high performance (Van De Schoot et al., 2021). The software displayed the titles and abstracts, and a reviewer decided whether each was relevant, exactly as in a traditional method. One of the challenges of using such software is determining when to consider all remaining articles as irrelevant and stop the process, balancing efficiency and accuracy. Following established practices (e.g., Ros et al., 2017), a stopping criterion was predetermined, where screening would end once enough articles were labelled irrelevant in a row. In this study, the stopping criterion was set at 100 papers. This process was conducted by one reviewer (HR) and resulted in 2033 articles being screened, with 634 labelled as initially relevant.

In any screening process, human error and screening fatigue are likely to lower accuracy. Following Brouwer et al. (2022), a procedure was therefore implemented to reduce the accidental exclusion of (potentially) relevant studies. First, a third reviewer (NH)^4^ was trained on the inclusion criteria using the same iterative process outlined above (round 1: agreement = 93%, kappa = 0.74; round 2: agreement = 95%, kappa = 0.87). Once training was complete, all records labelled as irrelevant (*n = *1399) or unlabelled (*n = *527) were re-entered into ASReview, with their labels now removed, and re-screened, using the same model but with 10 different articles as prior knowledge to provide a slightly different direction for the learning process. The same stopping criterion of 100 irrelevant papers in a row was applied, resulting in 1281 articles being screened and 51 articles reverted to the relevant pile. Thus, in total, 685 articles were deemed relevant for full-text screening.

**Figure 1.**
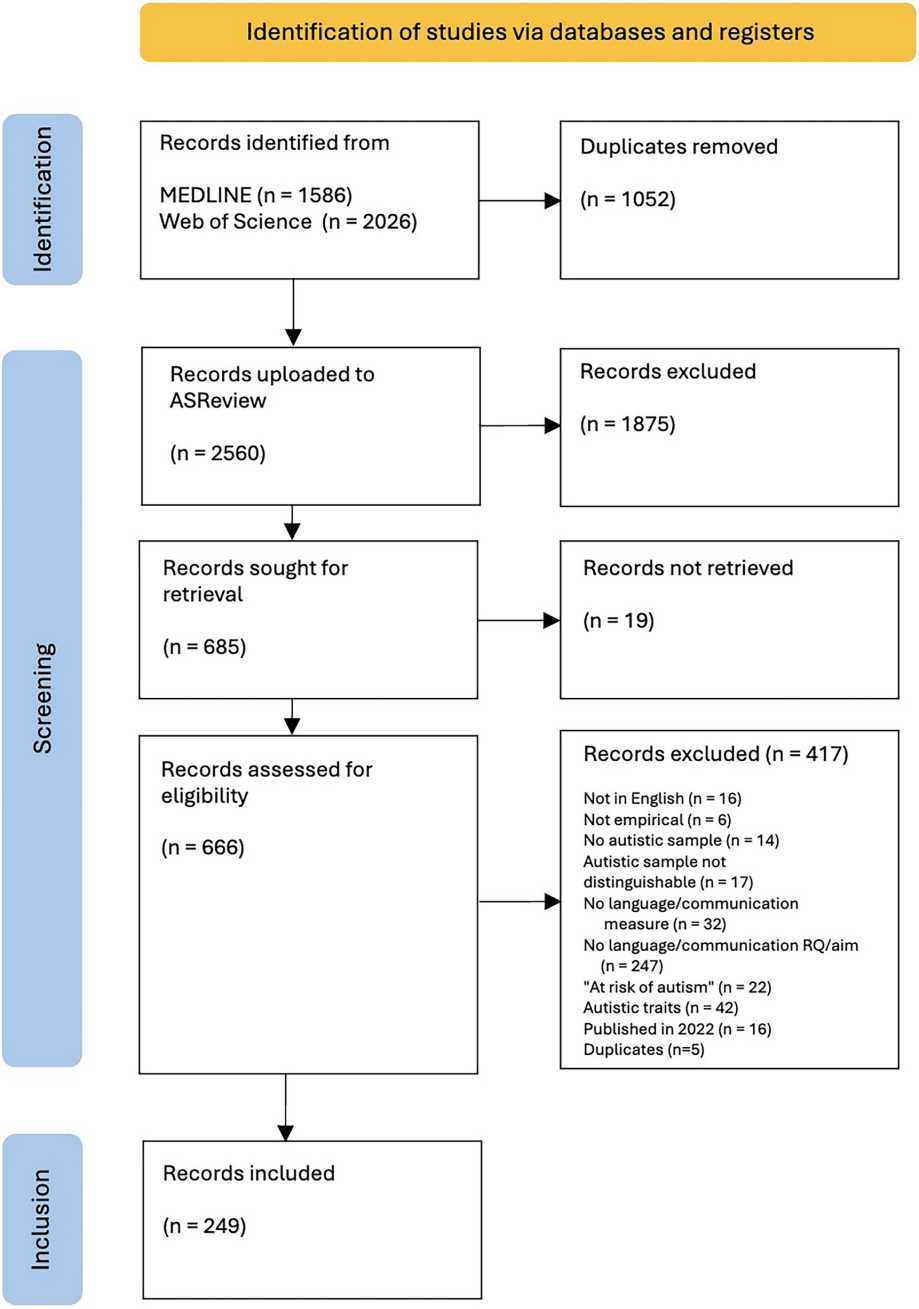
Identification, screening and inclusion process, based on PRISMA (see Page et al. 2021a).

In the full-text screening round, all criteria (see above) were applied and checked. Two reviewers (HR & NH) conducted an iterative process of blind double-coding to establish interrater reliability at this stage, the same as outlined above, but with a smaller sample size (*n = *35). Agreement in the first round was 89%, and Cohen's (unweighted) kappa was calculated as 0.83. As kappa was > 0.8, the process was not repeated. NH then individually screened 213 articles, and brought any articles where there was doubt forward for discussion. 21 articles were discussed with HR, of which 17 were resolved and 4 were further discussed between HR, TB and JV before being resolved. The remaining 472 articles were then screened by HR, with 19 discussed with TB and JV. After this final screening stage, 249 articles were included for coding (see Figure 1).

### Coding

Once the sample of 249 relevant articles was established, each article was individually reviewed and coded by one researcher (A1) on a number of aspects. This data can be found in the supplementary materials.

First, each article was coded according to a binary system (1/0) as to whether it included a neurotypical comparison group or not. We ignored comparisons to other neurodivergent groups (e.g., Specific Language Impairment) in this coding. Then, the text was searched for explicit hypotheses or predictions about how the groups would differ in performance. Explicitly made predictions were coded as (a) worse (i.e., the autistic group is predicted to score lower than the neurotypical group); (b) better, (c) both better and worse (i.e., on separate measures or accounting for both possibilities); (d) neither better nor worse (e.g., participants’ performance is predicted to be based on another factor besides group, such as a language score); or (e) no explicit prediction is made. Where multiple predictions were made within one study and this resulted in a combination of “neither” and either “better” or “worse” categories, the “better”/“worse” category was used. Again, we ignored any comparisons to other neurodivergent groups in this coding. This coding scheme and its limitations are commented on in the discussion section.

Second, studies were coded according to whether at least one participant in the sample was considered to have ID, resulting in five categories: (a) ID included; (b) ID excluded; (c) ambiguous ID inclusion; (d) no information about intellectual ability provided; or (e) participants are under the age of 2, in which case IQ is not yet reliably measurable.^5^ To categorise studies, the text was searched for explicit mention of ID inclusion/exclusion and/or the reporting of IQ scores. Where IQ scores were reported, the sample was considered to include ID if any participant scored <70 on a standardised measure (Russell et al., 2019), regardless of whether this was a full-scale IQ score or a specific measure, such as verbal IQ. If IQ scores were reported but without information as to whether any participant scored <70 (i.e., reporting group averages without range), this study was coded as being ambiguous in ID inclusion. Alternatively, if a study reported more than one measure of IQ (e.g., verbal and non-verbal IQ), and one measure indicated ID inclusion while the other did not, this was also coded as ambiguous, as were studies that reported measures other than IQ scores (e.g., developmental quotients or verbal age) that were difficult to interpret. In the discussion section of this paper, we comment on the limitations of this coding strategy.

Third, studies were also coded as to whether they included NMS participants. Due to the lack of standardisation in the literature surrounding NMS individuals, we coded any study that mentioned NMS (or “non-/minimally verbal”) participants, regardless of how they defined it, as well as related labels or descriptions, such as “lack of speech,” “severe language delay” etc. (for a full list, see the coding file in the supplementary materials). In the coding, we differentiated between (a) studies that explicitly included NMS participants; (b) those that did not explicitly mention inclusion (which may not necessarily mean exclusion); and (c) those studies that included participants under the age of 2, who are expected to be non- or minimally-speaking due to their age. Note that in the studies that did include NMS participants, this did not necessarily mean that the whole sample was NMS.

## Results

### Prevalence of Comparison Groups

Table 1 shows the proportion of studies with and without a neurotypical comparison group for each coded category (interventions, studies that include autistics with ID, and NMS inclusion). Overall, around half of the studies (49.4%) were conducted with a neurotypical comparison group. However, the use of such a comparison group was substantially contingent on the other coded aspects. Firstly, most studies (75.1%) in the sample were not interventions and neurotypical comparison groups were more common among non-intervention studies (63.6%) than among intervention studies (6.5%). Secondly, for ID, we can see that more studies excluded (28.1%) ID than included it (17.7%) and even more studies did not report information on this attribute (32.1%). In 14.5% of studies the inclusion of ID was ambiguous and among these studies there was a preference to compare autistic participants to neurotypicals (63.9%). Neurotypical comparison groups were more common among studies that explicitly excluded ID (80.0%) than among studies that explicitly included ID (34.1%). Where no information was provided about ID, studies often did not compare participants to neurotypicals, only doing so in 28.8% of cases. Finally, the majority of studies did not include NMS individuals (76.7%). Among the studies that did not include NMS individuals, the majority included a neurotypical comparison group (61.3%). In contrast, when NMS participants were included, no studies compared them to neurotypicals. Across the full sample, when looking at only non-interventions that did not include ID or NMS, the proportion of studies comparing autistic participants to neurotypicals is 75.4%.

**Table 1.** Overview of studies with and without neurotypical comparison group. ID = Intellectual Disability; NMS = Non-/Minimally Speaking. ^a^Note that percentage points do not add up to 100 for these categories as studies with participants <2 years old were removed from the sample for this overview (n=19).

	*Neurotypical comparison group (% of category)*	*No neurotypical comparison group (% of category)*	*Total in category (% of full sample)*
*Interventions*			
* Intervention*	4 (6.5%)	58 (93.5%)	62 (24.9%)
* Non-intervention*	119 (63.6%)	68 (36.4%)	187 (75.1%)
*Intellectual ability^a^*			
* Includes ID*	15 (34.1%)	29 (65.9%)	44 (17.7%)
* Excludes ID*	56 (80.0%)	14 (20.0%)	70 (28.1%)
* Ambiguous inclusion of ID*	23 (63.9%)	13 (36.1%)	36 (14.5%)
* No information about ID*	23 (28.8%)	57 (71.3%)	80 (32.1%)
*NMS^a^*			
* Includes NMS*	0 (0%)	39 (100%)	39 (15.7%)
* Does not include NMS*	117 (61.3%)	74 (38.7%)	191 (76.7%)
*Non-interventions that do not include ID or NMS*	98 (75.4%)	32 (24.6%)	130 (52.2%)
*Full sample*	123 (49.4%)	126 (50.6%)	249

ID = Intellectual Disability; NMS = Non-/Minimally Speaking. ^a^Note that percentage points do not add up to 100 for these categories as studies with participants <2 years old were removed from the sample for this overview (n=19).

### Predicting Autistic Performance

Table 2 takes data from all 123 studies that included a neurotypical comparison group and shows the proportion of studies that predicted the autistic group to perform worse, better, both better and worse, or neither better nor worse than the neurotypical group, or made no explicit prediction. From this, we see that a quarter of studies made no explicit prediction, but out of those that did make an explicit prediction (*n = *94), the majority (59.6%) expected the autistic group to perform worse.

**Table 2.** Predictions made about autistic versus neurotypical groups in studies that include a comparison group.

Prediction (autistic vs. neurotypical)	Studies
Worse	56 (45.5%)
Better	4 (3.3%)
Both	7 (5.7%)
Neither	27 (22.0%)
No prediction	29 (23.6%)
Total	123

### Intersections Between Interventions and Subpopulations

Table 3 shows the intersections between participant characteristics (ID and NMS) and whether a study is an intervention or not. In terms of intellectual ability, interventions were more common when ID was included (25.0%) than excluded (8.6%), but even more common when no information about ID was given (45.0%). Where ID inclusion was ambiguous, the proportion of interventions (16.7%) sits between the figures for inclusion and exclusion. Looking at this from another angle, most interventions (61.0%) did not include information about ID, and more included ID (18.6%) than excluded it (10.2%) or had ambiguous inclusion (10.2%). On the other hand, excluding ID was the more common (37.4%) for non-interventions than including ID (19.3%), ambiguous ID inclusion (17.5%) or providing no information about ID (25.7%). We can also see that 64.1% of studies involving NMS participants are intervention studies, while only 17.8% of studies that do not include NMS participants are interventions. Looking at this from another perspective, of all the non-intervention studies, the vast majority (91.8%) did not include NMS participants, while of all the intervention studies, only a small majority (57.6%) did not include NMS participants. In summary, participants with ID or who are NMS were overrepresented in intervention studies compared to non-interventions, and intervention studies were more likely to be conducted with ID or NMS subgroups than with groups that did not include ID or NMS participants.

**Table 3.** Intersections between intervention status and participant characteristics (NMS and intellectual ability). ID = Intellectual Disability; NMS = Non-/Minimally Speaking. ^a^Note that the total here does not match the total above for intervention/non-intervention studies because participants <2 years old were removed from the sample for this overview (n=19). ^b^The first percentage is calculated from the row total, while the second is calculated from the column total.

	Intervention	No intervention	Total
Intellectual ability			
* *Includes ID	11 (25.0%|18.6%)^b^	33 (75.0%|19.3%)	44
* *Excludes ID	6 (8.6%|10.2%)	64 (91.4%|37.4%)	70
* *Ambiguous ID inclusion	6 (16.7%|10.2%)	30 (83.3%|17.5%)	36
* *No Information about ID inclusion	36 (45.0%|61.0%)	44 (55.0%|25.7%)	80
NMS			
* *Includes NMS	25 (64.1%|42.4%)	14 (35.9%|8.2%)	39
* *Does not include NMS	34 (17.8%|57.6%)	157 (82.2%|91.8%)	191
Total^a^	59	171	

^a^Note that the total here does not match the total above for intervention/non-intervention studies because participants <2 years old were removed from the sample for this overview (*n* = 19). ^b^The first percentage is calculated from the row total, while the second is calculated from the column total.

Table 4 shows how the participant characteristics intellectual ability and NMS intersect with one another. Here we see that including ID was more common in studies where NMS participants were also included (25.0%) than not (17.3%). No studies excluded ID and included NMS participants, while 36.6% of studies that did not include NMS participants also excluded ID. Ambiguous ID inclusion was relatively similar regardless of whether NMS participants were included (16.7%) or not (15.7%), whereas it was more common to not give information about ID when NMS participants were included (56.4%) than not included (30.4%).

**Table 4.** Intersections between participant characteristics (intellectual ability and NMS). Note that studies with participants <2 years old are not included in this summary (n=19).

	Includes NMS	Does not include NMS	Total
Includes ID	11 (25.0%|28.2%)^a^	33 (75.0%|17.3%)	44
Excludes ID	0 (0%|0%)	70 (100%|36.6%)	70
Ambiguous ID Inclusion	6 (16.7%|15.4%)	30 (83.3%|15.7%)	36
No information ID Inclusion	22 (27.5%|56.4%)	58 (72.5%|30.4%)	80
Total	39	191	

^a^The first percentage is calculated from the row total, while the second is calculated from the column total.

A further breakdown of the data is given in the Appendix, where intersections between intervention status and participant characteristics (ID and NMS) are shown alongside whether a neurotypical comparison group was included. Due to the relatively low number of observations in some of the fields, this data is presented for completeness but is not further commented on.

## Discussion

This paper investigated the prevalence of comparative study designs across 249 studies into autistic language and communication published in 2023. It also assessed whether those studies that compared autistic participants to neurotypicals expected them to perform worse. We further explored whether patterns differed for interventions and studies that included intellectual disability (ID) or non-/minimally-speaking (NMS) participants, and how these characteristics intersected. Our results showed that across the full sample, roughly half of studies (49%) employed a comparative study design, and that this figure rose to 75% when looking at non-interventions that did not include ID or NMS participants. Of the 94 studies that did employ a comparative study design and did make an explicit prediction about group performance, 60% expected the autistic group to perform worse than their neurotypical peers, while only 4% expected a better performance. Taken together, these results show that there is a tendency for recent research in the field to be structured around comparing autistic individuals to neurotypical norms—unless the study is an intervention or includes participants that are already known to differ considerably from neurotypical levels of “functioning”—and that the purpose of such a comparison is often to discover autistic “deficits” in relation to the neurotypical ideal.

However, despite seeing this tendency within our sample, it is beyond the scope of our current investigation to say whether studies that do not compare autistics to neurotypicals are necessarily less norm-focused or more inclusive of neurodiversity. There are many factors involved in choosing a study design, and while we have attempted to explore some of these, in terms of interventions and subpopulations, there are presumably many other variables that may influence authors’ design choices. On the other hand, it is possible that a minority of studies that do compare autistic and neurotypicals may be couched in a neurodiversity-positive view (e.g., the 3% that expected autistics to perform better or the 6% that expected better and worse performance across different conditions). Importantly, what our results do show is that comparing autistics to the norm is normal within the field. Therefore, if neurodiversity is to be embraced by researchers, a critical examination of current methodology will be required.

Our study was, however, limited in scope by the one-year window that we analysed. This was necessary to keep the analysis feasible, and because we wanted to avoid limiting the scope in other ways, for example, by only considering a subfield within language and communication research. However, this short timeframe does limit the generalisability of our results and prevented us from analysing changes in practice over time.

In the remainder of this discussion, we elaborate on our findings regarding predictions of autistic performance, the use of intervention studies, and the inclusion of ID and NMS participants. For each of these, we discuss the limitations of our coding scheme and the scope of this study. We then close the discussion by outlining how future research can be approached from a less norm-based standpoint.

### Predicting Autistic Performance

Among the studies that used a comparative study design and made explicit predictions, the majority expected the autistic group to perform worse than their neurotypical peers. This shows, as has been discussed earlier (e.g., Chapman, 2020a), that empirical studies into autism are frequently based around the notion of an idealised neurotypical form of processing and positioning autistic individuals as less than this ideal, rather than investigating autistic experience or autistic ways of being in their own right. Although our data showed a strong trend towards negative predictions, there were limitations to our coding scheme which suggest that our analysis underrepresented the extent to which autism was negatively framed by study authors.

Firstly, we were rather conservative in our coding by only looking at how the autistic group was explicitly predicted to perform in comparison to the neurotypical group—specifically, we only looked at the section of each paper that outlined hypotheses and predictions. We did not, therefore, consider how autism was framed generally throughout the paper, even if certain predictions were implied elsewhere but not explicitly formulated. In total, a quarter of studies were coded as having no explicit predictions, and yet, there could be an implicit assumption that the autistic group would perform worse contained within the study aim or within theories and ideas of autistic “deficit.” For example, O’Brien et al. (2023) couch an assumption of worse autistic performance within their study's aim, by expecting to find “impairments”: “*The primary aim of this study was to identify the brain bases of phonological working memory impairments in autism.”* In another study, Ferguson et al. (2023) are similarly looking for “deficits,” but they do not clearly state that these are expected from the autistic group: “*As such, we compared PAQ scores across an ASD sample and a community control sample, to establish the extent to which deficits were present for specific alexithymia facets and/or specific emotional valences.*” From this, a reader familiar with the literature could logically assume that deficits were predominantly expected in the autistic group. These formulations therefore imply, to varying extents, that the authors are expecting worse performance from the autistic group. Yet, as these formulations were not made fully explicit in terms of a group comparison, they were not coded as making a “worse” prediction.

Other studies did not make an explicit prediction about group performance but did couch their research within theories that are deficit-based and/or have clear judgment values attached. Zarokanellou et al. (2023), for example, described the aim of their study as assessing “*two theoretical models—namely, the Poor Lexical-Semantic Structure Model and the Slow-Retrieval Model,*” both of which imply weakness. Similarly, some studies interpreted their results with a clear value-judgment attached, such as Leipold et al. (2023): “*Our findings support a social-cognitive deficit model of autism by identifying a role for TPJ dysfunction during emotional prosody processing.”* At both of these points—theoretical background and results interpretation—authors may highlight their assumptions and views about autism, but, once again, this was not captured by our coding procedure.

Another shortcoming with our coding scheme was that we were unable to unpack the implicit meanings behind neutral formulations, and which were therefore coded in the category of “neither worse nor better.” For example, Patel et al. (2023) made predictions based on “*acoustic markers of prosodic differences”* between autistic participants, their parents and controls. Based on this formulation alone, it is not clear whether such differences are positive, negative or truly neutral. However, elsewhere in the paper, “prosodic impairments” are mentioned, suggesting that this neutral formulation is in fact implicitly negative. In other cases, it was not clear whether the predicted effects were to be considered better or worse, for example in the case of brain imaging studies that predicted various increases or decreases in activation and/or brain volume. For example, Arutinian et al. (2023) wrote: “*for volume-based parameters, we hypothesized to detect either increased or decreased brain volume in ASD; for surface-based parameters, we expect to find increased GI, deeper SD, and abnormal FD in ASD. Additionally, we hypothesized that the atypical states of characteristics in language-related ROIs would be associated with language abilities of children with ASD.*” Interestingly, in this formulation, “atypical” or “abnormal” seems to be defined by whatever might be found within the autistic group. In any case, it is difficult, from this passage alone, to ascertain whether these descriptors are neutral. In the context of the wider paper though, which describes “pathological” brain volume and “impairments,” we can understand a negative implication, which once again fell outside of the scope of our coding.

So far, we have discussed cases where our coding did not capture pathology-driven thinking. Theoretically, the opposite could also be argued: our coding scheme may have failed to detect comparative studies that framed autism in a neuro-affirming manner. While this is possible, it is notable that none of the 29 studies coded as making no prediction explicitly mention neurodiversity theory. One of these studies did, however, centre autistic experience by examining how autistic and non-autistic children engaged with academic writing (Zajic et al., 2024). This paper, along with one other (Dunham et al., 2023), generally avoided potentially derogatory terms such as “disorder” or “deficit,” suggesting a more neuro-affirming stance than the other 27 papers that did use such language. As noted in the introduction, though, relying on language alone to assess framing is limited. Even in this small subsample, we see a mixing of language and ideas between paradigms. Huettig et al. (2023) describe autism as “a complex developmental neurodivergent phenomenon,” using neuro-affirming language, yet interpret their findings as indicating “a general deficit in visual attention,” drawing on pathology-based ideas. Conversely, Ochi et al. (2024) use terms such as “disorder” and “deficit” but justify their work in terms of improving quality of life. Taken together, these examples suggest that while our coding was also limited in detecting more nuanced neurodiversity-affirming approaches, such approaches appear rare in our sample.

Overall, while our results show quite clearly that comparative studies tended to explicitly predict autistic participants would perform worse than their neurotypical peers, our coding did not allow for a full exploration of how these assumptions can be found in other parts of the paper or in more implicit ways. As our coding scheme was rather strict, the numbers we presented for predicting worse autistic performance are likely an underestimation. This suggests that pathology-driven assumptions about autism might be even more dominant than our current results suggest. Similarly, a more nuanced analysis could shed light on how neurodiversity-affirming approaches are potentially emerging in the literature.

### Interventions

We started with the expectation that intervention studies would be less likely to include a neurotypical comparison group, due to the nature of intervention as a form of “remediation” for assumed “deficits.” This assumption is difficult to prove within the scope of our study, as we cannot tell the authors’ motivations for study design in this sense. However, we did notice that most intervention studies did not include neurotypicals. We now take a closer look at the studies in this category.

Of the four intervention studies (out of 62) that included a neurotypical control group, two focused on reading interventions (Kimhi et al., 2023; Muller et al., 2023), an area where neurotypical children can also benefit from explicit instruction, and therefore may be used to test intervention efficacy. This contrasts with, for example, teaching NMS individuals to match a picture with a specific request, where demonstrating that a neurotypical adult can do so adds little. The third study with a neurotypical comparison group involved a robot (Wang, 2023) and the fourth examined training syntax to improve theory of mind (Durrleman et al., 2023). In the robot study, the neurotypical control group partly functioned as a test of the robot itself, while for the theory of mind study, the neurotypical group helped show that manipulating one area of language (syntax) affected another skill (theory of mind). Thus, in all four cases, the control group was at least partly used to validate the intervention. This invites further reflection on why only a neurotypical group is treated as having this validating power. In some cases, there appears to be an ableist assumption that only neurotypicals can truly confirm whether an intervention “works.” This raises the question of whether, if an intervention were found to be effective for neurotypicals but not an autistic group, this finding would then be explained as evidence of autistic “impairment.”

Pathologising views may also be present in intervention studies that lack a neurotypical comparison group. A subset of papers in our intervention sample, 14 out of 62 studies, tested the effects of Applied Behaviour Analysis (ABA) therapy. ABA is a form of therapy that aims to improve the “functioning” of the autistic person through an intense programme of breaking behaviour down into discrete trials. It has been criticised by autistic advocates who have found the therapy harmful (Autism Self Advocacy Network, 2017), as well as academics who raise ethical concerns (Wilkenfeld & McCarthy, 2020). ABA therapy can therefore be considered a pathologising practice, but one that does not lend itself to comparative designs—of the 14 intervention studies that used this approach, only one (the robot study mentioned above) included a neurotypical comparison group. Thus, as we had anticipated, our measure of comparative study designs appears to be limited in capturing the pathologising nature within interventions.

Targeted interventions that genuinely address challenges and aim to improve quality of life for autistic individuals could be conducted, with or without a comparison group. We noted earlier that this is a priority for many autistic people and those who work with them (Cusack & Sterry, 2016). If improving quality of life is the goal and measure of success for such interventions, a comparison group is likely not necessary. However, we can also imagine a place for neuro-affirming comparison studies. For example, an intervention that targets (mis)communication between neurotypes from both directions (following Milton, 2012).

### Intellectual Ability

Turning our attention now to intellectual ability, we found that almost a third of studies in our sample did not report on this at all. This finding is similar to observations by Russell et al. (2019), who found that 38% of their 2016 sample of empirical studies about autism did not report intellectual ability. Where studies did include information about intellectual ability, we found that it was 1.6 times more common for studies to exclude than include ID. This trend is not particularly surprising, given that Russell et al. (2019) also found a sampling bias towards autistic participants without ID.

In terms of how intellectual ability interacted with the use of comparative study designs, two trends were visible. Firstly, we found that studies which included participants with ID tended to have no neurotypical comparison group, while the opposite was true for studies that excluded participants with ID, that is, they tended to have a neurotypical comparison group. This trend may result from two, not necessarily mutually exclusive, decision-making pathways in research design. On the one hand, if researchers have a general question about autism, they may opt for a comparative study as a first step in their design. From this point, and motivated by an attempt to “isolate” autism, researchers may wish to create two samples with as few differences between them as possible, and may therefore exclude participants with ID on this basis. This would then suggest that comparative studies being the default design^6^ leads to an underrepresentation of participants with ID. On the other hand, researchers who start the design process with a question about autism and ID may decide that a neurotypical comparison is not meaningful, either because too many differences would be expected, or because the question requires the use of a specific task only relevant for a population with ID (e.g., neurotypicals would score at ceiling level). As a second trend, we observed that the majority of studies without information about ID did not compare their sample to neurotypicals. This suggests that the main purpose of indicating that a sample excludes ID may be to show how comparable the sample is to neurotypical levels.

Within our study, a number of limitations became apparent during the coding of intellectual ability. For one, our criterion for whether a study included ID was rather inclusive, as we took any measure of IQ that dipped below 70 (see Russell et al., 2019) for any individual in the sample to be indicative of inclusion. This meant that some studies were considered to include ID where the autistic group range fell just below 70, perhaps indicating only one or a few participants in the sample had scores below this threshold. Frequently in these cases, the group mean was still comparable to a neurotypical control group, and so the authors may have treated the group as being without ID. Another issue with our coding was that we took any measure of IQ that was recorded below 70 as an indication of ID, regardless of whether this was a full IQ score or a subcomponent score, such as verbal or non-verbal IQ. It has previously been reported that autistic individuals frequently have an uneven IQ profile in terms of these subcomponents, with verbal IQ scores typically being lower than non-verbal scores (Grondhuis et al., 2018). Therefore, if the study authors only reported verbal IQ, there was presumably a higher likelihood of us considering the sample to include ID than if only non-verbal IQ was reported. This bias became particularly apparent in two studies where both verbal and non-verbal IQ were reported as individual scores, and where we found, according to our criteria, verbal scores indicated ID inclusion, and non-verbal scores indicated ID exclusion (we coded such cases as “ambiguous”). Other studies (also coded as “ambiguous”) reported mean IQ scores, but without stating the range, so that it was not possible to tell if scores below 70 were included. It is worth noting that in these studies, IQ was typically reported as a group mean to show that autistic and neurotypical participants did not differ in intellectual ability, at least on the group level. Thus, we found instances where study authors treated the sample as though it did not include ID, whereas our coding erred on the more conservative side by placing these studies in the “ambiguous” category. Finally, it is of course notable that any form of inclusion/exclusion that relies on a linear scale with an arbitrary cut-off point is imperfect as it creates strict divisions where reality is more fluid (see e.g., Greenspan et al., 2015), and many of the limitations of our categorical coding scheme stem from this underlying imperfection.

### NMS

We predicted that studies that included NMS participants would be less likely to use a neurotypical control group as this subpopulation is already known to differ from the “norm” in terms of spoken language use. Strikingly, no studies reported including NMS participants as well as a neurotypical comparison group, in line with our prediction.

Within our sample there was a noticeable overlap between studies investigating NMS individuals and performing an intervention. This shows that there is a tendency to view the NMS population as a group that needs “remediation.” While there may be well-meaning intentions behind such interventions, we may wish to question whether they are centred around norm-based assumptions of communication, and whether this is the most helpful approach. An alternative perspective would be to consider how NMS individuals *do* communicate, and to build research practices from this foundation (see Aidonopoulou-Read, 2025 for a framework). In this way, studies could be more aligned with improving quality of life within the NMS population, rather than achieving certain goals that may be based on external expectations (Evers et al., 2022; Higashida, 2022).

Finally, we can see from our results that there is a noteworthy overlap between ID and NMS inclusion. In our sample, a quarter of the studies that included ID also included NMS participants, while no studies reported excluding ID and including NMS participants. This would seem to imply that typically NMS people additionally have ID, which has been noted as a frequent, but questionable, assumption in the literature (Dawson et al., 2007). Administering IQ tests to NMS participants and interpreting the results are challenging endeavours (Kasari et al., 2013), and questions may therefore be raised as to how valid, and how useful, ID labels may be within the NMS population.

### Future Directions for the Field

If norm-centred comparative studies should be limited, the question thus arises as to where research should be directed instead. A neurodiversity-positive research agenda would frequently start with a participatory research approach, where autistic individuals help design and shape research according to their needs (Ayaya, 2023; Fletcher-Watson et al., 2025). Researchers might also start by considering common autistic experiences, such as sensory challenges (see e.g., Sibeoni et al., 2022; Taels et al., 2023), social exclusion (see e.g., Jones et al., 2022) or minority stress (see e.g., Botha & Frost, 2020) and investigate how these experiences influence learning language and communicating effectively. Alternatively, research could involve surveying autistic participants about the real-world communicative problems they face, such as difficulties processing speech-in-noise (e.g., Sturrock et al., 2022) and investigate strategies and accommodations for such challenges. Another avenue is starting with the assumption that any miscommunication is a two-way problem between individuals (i.e., in line with the Double Empathy Problem, Milton, 2012, and the social model of disability, Oliver, 1983, 2013), thus investigating instances of miscommunication between autistics and neurotypicals from both sides (e.g., Crompton et al., 2020; Edey et al., 2016). Most importantly, although neurotypicals may still be implicated in some areas of autistic research (e.g., the ways in which they discriminate against or miscommunicate with autistics), a neurodiversity-positive autistic research programme will replace notions of neurotypical expectations with autistic experience as its centrepiece.

## Conclusion

Our systematic and critical cross-sectional review of autistic language and communication research from 2023 showed a tendency for empirical studies to be based around norm comparisons, where autistic individuals are expected to perform worse than their neurotypical counterparts. Our study also showed that some study designs, namely interventions, as well as studies that included participants with ID or who were NMS followed a different pattern, where comparing to neurotypicals was less likely. We have suggested that this is because interventions are frequently designed to “normalise” rather than to investigate the extent to which the population is “normal,” and because individuals with ID or who are NMS are already presumed to differ “too much” from “normal” expectations. While it was beyond the scope of our current investigation to fully elaborate on the connection between comparative study designs and pathology-driven thinking, our results show that such studies make up a core of the current empirical research into autistic language and communication, and may be considered within the field to be a marker of standard or “good” methodology. If the predicted neurodiversity paradigm shift (Manalili et al., 2023; Pellicano & den Houting, 2022) is to take place, the structure of the current methodological landscape will presumably need to shift away from such norm-based traditions.

## Supplemental Material

sj-xlsx-1-dli-10.1177_23969415261441522 - Supplemental material for Comparing to a Neurotypical Norm Is Normal in Autistic Language and Communication Research: A Cross-Sectional Systematic Review and Critical Analysis of Recent Literature Supplemental material, sj-xlsx-1-dli-10.1177_23969415261441522 for Comparing to a Neurotypical Norm Is Normal in Autistic Language and Communication Research: A Cross-Sectional Systematic Review and Critical Analysis of Recent Literature by Harrie Reynolds, Titia Benders and Josje Verhagen in Autism & Developmental Language Impairments

sj-docx-2-dli-10.1177_23969415261441522 - Supplemental material for Comparing to a Neurotypical Norm Is Normal in Autistic Language and Communication Research: A Cross-Sectional Systematic Review and Critical Analysis of Recent Literature Supplemental material, sj-docx-2-dli-10.1177_23969415261441522 for Comparing to a Neurotypical Norm Is Normal in Autistic Language and Communication Research: A Cross-Sectional Systematic Review and Critical Analysis of Recent Literature by Harrie Reynolds, Titia Benders and Josje Verhagen in Autism & Developmental Language Impairments

## Notes

1. Note that terminology related to the medical model is placed in quotation marks throughout the text.

2. A reference to the nonsensical poem *The Hunting of the Snark* by Lewis Carroll (1876).

3. Note that here we employ this terminology rather than the typically used “nonverbal” or “minimally verbal” because “non-speaking” and “minimally speaking” are preferred by those who these terms designate (Zisk & Konyn, 2022), and are arguably more accurate descriptors (Bottema-Beutel et al., 2025).

4. Nora Harvei, rMA student at UvA.

5. For example, the Stanford-Binet Intelligence Scale is especially designed for use with young children and starts from age 2 (Roid, 2003).

6. The preference towards comparative studies may be to some extent implicit, however some explicit references may also be found. For example, the Journal of Autism and Developmental Disorders (JADD) states in their guidelines under “types of paper”: “JADD is no longer accepting manuscripts with only one participant or group studies without an appropriate comparison group” (JADD, n.d.).

## Appendix

[Table table5-23969415261441522] [Table table6-23969415261441522]

**Table A1. table5-23969415261441522:** The Intersections Between Intervention Status, Participant Characteristics (ID and NMS) and Whether a Neurotypical Comparison Group Was Included.

	Intervention			No Intervention		
	Comparison group	No Comparison group	Total	Comparison group	No Comparison group	Total
NMS						
Includes NMS	0	25	25	0	14	14
Does not include NMS	4	30	34	113	44	157
Intellectual ability						
Includes ID	0	11	11	15	18	33
Excludes ID	2	4	6	54	10	64
Ambiguous ID inclusion	0	6	6	23	7	30
No information ID inclusion	2	34	36	21	23	44
Total	4	55		113	58	

**Table A2. table6-23969415261441522:** The Intersections Between Participant Characteristics (ID and NMS) and Whether a Neurotypical Comparison Group Was Included.

	Includes NMS			Does not include NMS		
	Comparison group	No comparison group	Total	Comparison group	No comparison group	Total
Includes ID	0	11	11	15	18	33
Excludes ID	0	0	0	56	14	70
Ambiguous ID inclusion	0	6	6	23	7	30
No information ID inclusion	0	22	22	23	35	58
Total	0	39		117	74	
