# Supplementary material for: Comparing to a Neurotypical Norm Is Normal in Autistic Language and Communication Research: A Cross-Sectional Systematic Review and Critical Analysis of Recent Literature
Source: Autism Dev Lang Impair. 2026 May 24;11:23969415261441522. doi: 10.1177/23969415261441522 (PMC13199701; doi:10.1177/23969415261441522)
Supplement: sj-docx-2-dli-10.1177_23969415261441522 - Supplemental material for Comparing to a Neurotypical Norm Is Normal in Autistic Language and Communication Research: A Cross-Sectional Systematic Review and Critical Analysis of Recent Literature [file sj-docx-2-dli-10.1177_23969415261441522.docx]

**Supplementary Materials: Search Terms and Studies Reference List**

**Search Terms**

| **Search** | **Target** | **MEDLINE Syntax** | **Web Of Science Syntax** |
| --- | --- | --- | --- |
| #1 | Autism | Autism spectrum disorder/ OR autistic disorder/ OR asperger syndrome/ OR (autis* OR ASD OR asperger* OR pervasive developmental disorder* OR PDD-NOS).ti,ab,kf. | TS=("autis*" OR "ASD" OR "asperger*" OR "pervasive developmental disorder*" OR "PDD-NOS") |
| #2 | Language/ Communication | (languag* OR (communicat* ADJ3 (social* OR socio* OR difficult* OR skill* OR autis* OR deficit* OR barrier* OR interpersonal OR *verbal* OR disorder*)) OR speech OR verbal* OR vocal* OR linguistic* OR pragmatic* OR phonolog* OR phonetic* OR semantic* OR synta* OR morpholog* OR morphosynta* OR morpho-synta* OR lexic* OR gestur* OR multimodal*).ti,ab,kf,hw. | TS=("languag*" OR ("communicat*" NEAR/2 ("social*" OR “socio*” OR "difficult*" OR "skill*" OR "autis*" OR "deficit*" OR "barrier*" OR "interpersonal" OR "*verbal*" OR "disorder*")) OR "speech" OR "verbal*" OR "vocal*" OR "linguistic*" OR "pragmatic*" OR "phonolog*" OR "phonetic*" OR "semantic*" OR "synta*" OR "morpholog*" OR "morphosynta*" OR “morpho-synta*” OR "lexic*" OR “gestur*” OR “multimodal*”) |
| #3 | Study Type | cohort studies/ OR follow-up studies/ OR longitudinal studies/ OR prospective studies/ OR retrospective studies/ OR controlled before-after studies/ OR cross-sectional studies/ OR randomized controlled trial/ OR (cohort* OR cross-sectional OR followup OR follow-up OR longitud* OR pretest* OR posttest* OR pre-test* OR post-test* OR pre-treatment OR post-treatment OR pretreatment OR posttreatment OR prospectiv* OR quasi-exp* OR quasiexp* OR repeated measur* OR retrospectiv* OR control group* OR control condition* OR control design* OR random* OR rct* OR controlled trial* OR controlled study OR experimental study OR experimental design* OR treatment group* OR waiting list OR waitlist OR auc OR cohen*s d OR correlat* OR covariat* OR effect size OR odds ratio OR linear regression OR logistic regression OR inter-rater reliability OR interrater reliability OR anteced* OR factor* OR mediator* OR moderater* OR moderating OR moderator* OR path? OR pathway* OR predictor* OR variable* OR direct effect? OR interaction effect? OR indirect effect? OR main effect? OR negative effect? OR positive effect? OR significant effect? OR significant result? OR significant differ* OR instrument OR checklist* OR inventory OR questionnair* OR rating* OR scale* OR self-report* OR survey OR task* OR test* OR associated OR baseline OR empirical* OR experiment* OR design* OR outcomes OR participant* OR related with OR sample OR data OR discourse anal* OR themat* synthes* OR frame* OR ethnograph* OR interlocut* OR case stud*).ti,ab,kf. | TS=("cohort*" OR "cross-sectional" OR "followup" OR "follow-up" OR "longitud*" OR "pretest*" OR "posttest*" OR "pre-test*" OR "post-test*" OR "pre-treatment" OR "post-treatment" OR "pretreatment" OR "posttreatment" OR "prospectiv*" OR "quasi-exp*" OR "quasiexp*" OR "repeated measur*" OR "retrospectiv* " OR "control group*" OR "control condition*" OR "control design*" OR "random*" OR "rct*" OR "controlled trial*" OR "controlled study" OR "experimental study" OR "experimental design*" OR "treatment group*" OR "waiting list" OR "waitlist" OR "auc" OR "cohen*s d" OR "correlat*" OR "covariat*" OR "effect size" OR "odds ratio" OR "linear regression" OR "logistic regression" OR "inter-rater reliability" OR "interrater reliability" OR "anteced*" OR "factor*" OR "mediator*" OR "moderater*" OR "moderating" OR "moderator*" OR "path$" OR "pathway*" OR "predictor*" OR "variable*" OR "direct effect$" OR "interaction effect$" OR "indirect effect$" OR "main effect$" OR "negative effect$" OR " positive effect$" OR "significant effect$" OR "significant result$" OR "significant differ*" OR "instrument" OR "checklist*" OR "inventory" OR "questionnair*" OR "rating*" OR "scale*" OR "self-report*" OR "survey" OR "task*" OR "test*" OR "associated" OR "baseline" OR "empirical*" OR "experiment*" OR "design*" OR "outcomes" OR "participant*" OR "related with" OR "sample" OR "data" OR "discourse anal*" OR "themat* synthes*" OR "frame*" OR "ethnograph*" OR "interlocut*" OR "case stud*") |
| #4 | (Non-)Human Subjects | animal/ NOT human/ | TS=(("animal*" OR nonhuman OR "mammal*" OR "guinea pig*" OR "monkey*" OR "mouse" OR "mice" OR "rabbit*" OR "rat" OR "rats" OR "rodent*" OR "dog" OR "dogs" OR "cat" OR "cats" OR "chicken*") NOT ("human" OR "humans" OR "participant*" OR "subject" OR "subjects" OR "patient" OR "patients" OR "men*" OR "male" OR "males" OR "wom*n" OR "female*" OR "child*" OR "adolescen*" OR "toddler*" OR "human" OR "humans" OR "participant*" OR "subject" OR "subjects" OR "patient" OR "patients" OR "men*" OR "male" OR "males" OR "wom*n" OR "female*" OR "child*" OR "adolescen*" OR "teen*" OR "youth*" OR "young*" OR "toddler*" OR "infan*" OR "boy*" OR "girl*" OR "people" OR "individual*" OR "student*" OR "preschool*" OR "adult*" OR "elderly")) |
| #5 | Autism & Language/ Communication & Study Type | #1 AND #2 AND #3 | #1 AND #2 AND #3 |
| #6 | Autism & Language/ Communication & Study Type & Human Subjects | #5 NOT #4 | #5 NOT #4 |

Table S1: Terms used for database searches

**Reference List of Included Studies**

Abdi, S., Tarameshlu, M., Nakhostin Ansari, N., Ghelichi, L., & Hakim Shooshtari, M. (2023). The Effect of Combined Intervention on Improvement of Early Lexical Development in Minimally Verbal Children with Autism Spectrum Disorder. *Medical Journal of The Islamic Republic of Iran*. <https://doi.org/10.47176/mjiri.37.104>

Adornetti, I., Chiera, A., Altavilla, D., Deriu, V., Marini, A., Gobbo, M., Valeri, G., Magni, R., & Ferretti, F. (2024). Defining the Characteristics of Story Production of Autistic Children: A Multilevel Analysis. *Journal of Autism and Developmental Disorders*, *54*(10), 3759–3776. <https://doi.org/10.1007/s10803-023-06096-2>

Alho, J., Khan, S., Mamashli, F., Perrachione, T. K., Losh, A., McGuiggan, N. M., Graham, S., Nayal, Z., Joseph, R. M., Hämäläinen, M. S., Bharadwaj, H., & Kenet, T. (2023). Atypical cortical processing of bottom-up speech binding cues in children with autism spectrum disorders. *NeuroImage: Clinical*, *37*, 103336. <https://doi.org/10.1016/j.nicl.2023.103336>

Alho, J., Samuelsson, J. G., Khan, S., Mamashli, F., Bharadwaj, H., Losh, A., McGuiggan, N. M., Graham, S., Nayal, Z., Perrachione, T. K., Joseph, R. M., Stoodley, C. J., Hämäläinen, M. S., & Kenet, T. (2023). Both stronger and weaker cerebro‐cerebellar functional connectivity patterns during processing of spoken sentences in autism spectrum disorder. *Human Brain Mapping*, *44*(17), 5810–5827. <https://doi.org/10.1002/hbm.26478>

Alhozyel, E., Elbedour, L., Balaum, R., Meiri, G., Michaelovski, A., Dinstein, I., Davidovitch, N., Kerub, O., & Menashe, I. (2023). Association Between Early Developmental Milestones and Autism Spectrum Disorder. *Research on Child and Adolescent Psychopathology*, *51*(10), 1511–1520. <https://doi.org/10.1007/s10802-023-01085-6>

Alon-Tirosh, M., & Meir, N. (2023). Use of social network sites among adolescents with autism spectrum disorder: A qualitative study. *Frontiers in Psychology*, *14*, 1192475. <https://doi.org/10.3389/fpsyg.2023.1192475>

Alvarado, Y., Guerrero, R., & Serón, F. (2023). Inclusive Learning through Immersive Virtual Reality and Semantic Embodied Conversational Agent: A case study in children with autism. *Journal of Computer Science and Technology*, *23*(2), e09. <https://doi.org/10.24215/16666038.23.e09>

Alvarez, C. F., & Wolfe, B. H. (2024). Examining the face threatening acts individuals with autism spectrum disorder experience and how they respond. *Journal of Social and Personal Relationships*, *41*(1), 274–301. <https://doi.org/10.1177/02654075231210029>

Apeksha, K., Hanasoge, S., Jain, P., & Babu, S. S. (2023). Speech Perception in Quiet and in the Presence of Noise in Children with Autism Spectrum Disorder: A Behavioral Study. *Indian Journal of Otolaryngology and Head & Neck Surgery*, *75*(3), 1707–1711. <https://doi.org/10.1007/s12070-023-03721-5>

Artis, J., & Arunachalam, S. (2023). Semantic and Syntactic Properties of Words and the Receptive–Expressive Gap in Autistic and Non-Autistic Children. *Journal of Speech, Language, and Hearing Research*, *66*(5), 1771–1791. <https://doi.org/10.1044/2023_JSLHR-22-00369>

Artuso, C., & Belacchi, C. (2023). Implicit Grammatical Gender Representation in Italian Children with Autism without Intellectual/Language Disorder. *Children*, *10*(11), 1737. <https://doi.org/10.3390/children10111737>

Arutiunian, V., Arcara, G., Buyanova, I., Davydova, E., Pereverzeva, D., Sorokin, A., Tyushkevich, S., Mamokhina, U., Danilina, K., & Dragoy, O. (2023). Neuromagnetic 40 Hz Auditory Steady-State Response in the left auditory cortex is related to language comprehension in children with Autism Spectrum Disorder. *Progress in Neuro-Psychopharmacology and Biological Psychiatry*, *122*, 110690. <https://doi.org/10.1016/j.pnpbp.2022.110690>

Arutiunian, V., Gomozova, M., Minnigulova, A., Davydova, E., Pereverzeva, D., Sorokin, A., Tyushkevich, S., Mamokhina, U., Danilina, K., & Dragoy, O. (2023). Structural brain abnormalities and their association with language impairment in school-aged children with Autism Spectrum Disorder. *Scientific Reports*, *13*(1), 1172. <https://doi.org/10.1038/s41598-023-28463-w>

Atherkode, S., & Mason, L. (2024). Assessing the Verbal Behavior of a Linguistically Diverse Speaker with Autism. *The Analysis of Verbal Behavior*, *40*(2), 271–279. <https://doi.org/10.1007/s40616-023-00196-x>

Barsotti, J., Mangani, G., Nencioli, R., Narzisi, A., Pfanner, L., Chilosi, A. M., Cipriani, P., Mancini, A., Cosenza, A., Tancredi, R., & Calderoni, S. (2023). Sex/Gender Differences in the Language Profiles of Italian Children with Autism Spectrum Disorder: A Retrospective Study. *Journal of Clinical Medicine*, *12*(15), 4923. <https://doi.org/10.3390/jcm12154923>

Bateman, K. J., Wilson, S. E., Ingvarsson, E., Doucette, J., Therrien, W., Nevill, R., & Mazurek, M. (2023). Snack Talk: Effects of a Naturalistic Visual Communication Support on Increasing Conversation Engagement for Adults with Disabilities. *Behavior Analysis in Practice*, *16*(4), 1085–1099. <https://doi.org/10.1007/s40617-023-00775-3>

Beauchamp, M. L. H., Rezzonico, S., Bennett, T., Duku, E., Georgiades, S., Kerns, C., Mirenda, P., Richard, A., Smith, I. M., Szatmari, P., Vaillancourt, T., Waddell, C., Zaidman-Zait, A., Zwaigenbaum, L., & Elsabbagh, M. (2023). The Influence of Bilingual Language Exposure on the Narrative, Social and Pragmatic Abilities of School-Aged Children on the Autism Spectrum. *Journal of Autism and Developmental Disorders*, *53*(12), 4577–4590. <https://doi.org/10.1007/s10803-022-05678-w>

Benitez, P., & Domeniconi, C. (2023). Equivalence‐based instruction to teaching reading by families and teachers students with autism and/or intellectual disabilities. *Behavioral Interventions*, *38*(3), 861–880. <https://doi.org/10.1002/bin.1932>

Bergmann, S., Niland, H., Otero, M., Gavidia, V. L., & Kodak, T. (2023). Teaching children with autism spectrum disorder to tact auditory stimuli: A replication. *Behavioral Interventions*, *38*(3), 636–652. <https://doi.org/10.1002/bin.1936>

Birkeneder, S. L., & Sparapani, N. (2023). Measurements of Spontaneous Communication Initiations in Children with Autism in Preschool through Third Grade Classrooms. *Journal of Autism and Developmental Disorders*, *53*(3), 1243–1254. <https://doi.org/10.1007/s10803-022-05738-1>

Birri, N. L., Carnahan, C. R., Schmidt, C., & Williamson, P. (2023). A Personal Narrative Intervention for Adults With Autism and Intellectual Disability. *American Journal on Intellectual and Developmental Disabilities*, *128*(1), 21–35. <https://doi.org/10.1352/1944-7558-128.1.21>

Briend, F., David, C., Silleresi, S., Malvy, J., Ferré, S., & Latinus, M. (2023). Voice acoustics allow classifying autism spectrum disorder with high accuracy. *Translational Psychiatry*, *13*(1), 250. <https://doi.org/10.1038/s41398-023-02554-8>

Broome, K., McCabe, P., Docking, K., Doble, M., & Carrigg, B. (2023). Speech Development Across Subgroups of Autistic Children: A Longitudinal Study. *Journal of Autism and Developmental Disorders*, *53*(7), 2570–2586. <https://doi.org/10.1007/s10803-022-05561-8>

Butler, L. K., Shen, L., Chenausky, K. V., La Valle, C., Schwartz, S., & Tager-Flusberg, H. (2023). Lexical and Morphosyntactic Profiles of Autistic Youth With Minimal or Low Spoken Language Skills. *American Journal of Speech-Language Pathology*, *32*(2), 733–747. <https://doi.org/10.1044/2022_AJSLP-22-00098>

Butler, L. K., & Tager‐Flusberg, H. (2023). Fine motor skill and expressive language in minimally verbal and verbal school‐aged autistic children. *Autism Research*, *16*(3), 630–641. <https://doi.org/10.1002/aur.2883>

Bylemans, T., Heleven, E., Asselman, E., Baetens, K., Deroost, N., Baeken, C., & Van Overwalle, F. (2023). Sex differences in autistic adults: A preliminary study showing differences in mentalizing, but not in narrative coherence. *Acta Psychologica*, *236*, 103918. <https://doi.org/10.1016/j.actpsy.2023.103918>

Cairney, B. E., West, S. H., Haebig, E., Cox, C. R., & Lucas, H. D. (2023). Interpretations of meaningful and ambiguous hand gestures in autistic and non-autistic adults: A norming study. *Behavior Research Methods*, *56*(5), 5232–5245. <https://doi.org/10.3758/s13428-023-02268-1>

Camero, R., Gallego, C., & Martínez, V. (2024). Gaze Following as an Early Diagnostic Marker of Autism in a New Word Learning Task in Toddlers. *Journal of Autism and Developmental Disorders*, *54*(9), 3211–3224. <https://doi.org/10.1007/s10803-023-06043-1>

Caron, J., Light, J., & McNaughton, D. (2023). Effects of adapted Letter-Sound correspondence instruction with older learners with complex communication needs and autism spectrum disorder. *Augmentative and Alternative Communication*, *39*(1), 45–59. <https://doi.org/10.1080/07434618.2022.2121226>

Carruthers, S., Pickles, A., Charman, T., McConachie, H., Le Couteur, A., Slonims, V., Howlin, P., Collum, R., Salomone, E., Tobin, H., Gammer, I., Maxwell, J., Aldred, C., Parr, J., Leadbitter, K., & Green, J. (2024). Mediation of 6‐year mid‐childhood follow‐up outcomes after pre‐school social communication (PACT) therapy for autistic children: Randomised controlled trial. *Journal of Child Psychology and Psychiatry*, *65*(2), 233–244. <https://doi.org/10.1111/jcpp.13798>

Caruana, N., Nalepka, P., Perez, G. A., Inkley, C., Munro, C., Rapaport, H., Brett, S., Kaplan, D. M., Richardson, M. J., & Pellicano, E. (2024). Autistic young people adaptively use gaze to facilitate joint attention during multi-gestural dyadic interactions. *Autism*, *28*(6), 1565–1581. <https://doi.org/10.1177/13623613231211967>

Castellón, F. A., Sturm, A., & Kasari, C. (2024). Dual Identification: Trajectories to English Proficiency for English Learners with Autism Spectrum Disorder. *Journal of Autism and Developmental Disorders*, *54*(7), 2615–2624. <https://doi.org/10.1007/s10803-023-05994-9>

Chan, A. S., Ding, Z., Lee, T., Sze, S. L., & Cheung, M.-C. (2023). Temporal processing deficit in children and adolescents with autism spectrum disorder: An online assessment. *DIGITAL HEALTH*, *9*, 20552076231171500. <https://doi.org/10.1177/20552076231171500>

Chen, A., Zhao, R., Huang, G., Li, A., & Cheung, H. (2023). Successful lexical tone production of Mandarin Chinese autistic children with intellectual impairment. *International Journal of Language & Communication Disorders*, *58*(6), 1912–1926. <https://doi.org/10.1111/1460-6984.12881>

Chen, L., He, X., & Durrleman, S. (2023). Acquisition of grammatical aspect by Mandarin‐speaking preschool children with autism spectrum disorder. *International Journal of Language & Communication Disorders*, *58*(5), 1697–1716. <https://doi.org/10.1111/1460-6984.12897>

Chen, X., & Huang, A. X. (2025). Training Asian Immigrant Parents to Teach Manding to Children with Autism Spectrum Disorder. *Journal of Behavioral Education*, *34*(1), 184–212. <https://doi.org/10.1007/s10864-023-09526-4>

Chen, Y., Siles, B., & Tager‐Flusberg, H. (2024). Receptive language and receptive‐expressive discrepancy in minimally verbal autistic children and adolescents. *Autism Research*, *17*(2), 381–394. <https://doi.org/10.1002/aur.3079>

Chladek, M., Burbridge, C., Gibbons, E., Willgoss, T., Smith, J., & Clinch, S. (2023). Qualitative Exploration in Exit Interviews of Changes Observed in Clinical Trials for Individuals with Autism Spectrum Disorder Without Intellectual Disability. *Patient Related Outcome Measures*, *Volume 14*, 313–335. <https://doi.org/10.2147/PROM.S385682>

Cho, S., Cola, M., Knox, A., Pelella, M. R., Russell, A., Hauptmann, A., Covello, M., Cieri, C., Liberman, M., Schultz, R. T., & Parish-Morris, J. (2023). Sex differences in the temporal dynamics of autistic children’s natural conversations. *Molecular Autism*, *14*(1), 13. <https://doi.org/10.1186/s13229-023-00545-6>

Clark, G. T., & Reuterskiöld, C. (2023). Word Learning With Orthographic Support in Nonspeaking and Minimally Speaking School-Age Autistic Children. *Journal of Speech, Language, and Hearing Research*, *66*(6), 2047–2063. <https://doi.org/10.1044/2023_JSLHR-22-00549>

Clarke, K. A., Siegel, M., & Williams, D. L. (2023). The Relationship Between Augmentative and Alternative Communication Use by Pediatric Psychiatric Inpatients With Autism Spectrum Disorder and Interfering Behaviors. *American Journal of Speech-Language Pathology*, *32*(5), 2040–2056. <https://doi.org/10.1044/2023_AJSLP-23-00019>

Clin, E., & Kissine, M. (2023). Listener- Versus Speaker-Oriented Disfluencies in Autistic Adults: Insights From Wearable Eye-Tracking and Skin Conductance Within a Live Face-to-Face Paradigm. *Journal of Speech, Language, and Hearing Research*, *66*(8), 2562–2580. <https://doi.org/10.1044/2023_JSLHR-23-00002>

Coburn, K. L., & Williams, D. L. (2023). Quantitative Analysis of Narrative Discourse by Autistic Adults of Underrepresented Genders. *Autism in Adulthood*, *5*(2), 154–164. <https://doi.org/10.1089/aut.2021.0080>

Cohen, S. R., Wishard Guerra, A., Miguel, J., Bottema-Beutel, K., & Oliveira, G. (2025). *Hablando* at home: Examining the interactional resources of a bilingual autistic child. *Journal of Child Language*, *52*(1), 135–157. <https://doi.org/10.1017/S0305000923000600>

Cohn, E. G., Harrison, M. J., & McVilly, K. R. (2024). ‘Let me tell you, I see echolalia as being a part of my son’s identity’: Exploring echolalia as an expression of neurodiversity from a parental perspective. *Autism*, *28*(5), 1245–1257. <https://doi.org/10.1177/13623613231195795>

Cole, C. L., Bambara, L. M., Telesford, A. E., Bauer, K., Bilgili-Karabacak, I., Chovanes, J., Thomas, A., & Weir, A. (2023). Using a Brief Intervention to Improve Partner-Focused Conversation in Adolescents with Autism. *Journal of Autism and Developmental Disorders*, *53*(6), 2203–2218. <https://doi.org/10.1007/s10803-022-05510-5>

Conine, D. E., Guerrero, L. A., Jones-Thomas, E., Frampton, S. E., Vollmer, T. R., & Smith-Bonahue, T. (2023). Verbal Behavior Analysis of Teaching Story Recall to Children with Autism: A Replication and Extension. *The Analysis of Verbal Behavior*, *39*(1), 118–145. <https://doi.org/10.1007/s40616-023-00183-2>

Crutcher, J., Butler, E., Burke, J. D., Naigles, L., Fein, D. A., & Eigsti, I.-M. (2023). Pragmatic language and associations with externalizing behaviors in autistic individuals and those who have lost the autism diagnosis. *Research in Autism Spectrum Disorders*, *108*, 102252. <https://doi.org/10.1016/j.rasd.2023.102252>

Da Cruz, F. M. (2023). Multimodal interaction analysis of non-lexical vocalisations in low-verbal autistic children. *Clinical Linguistics & Phonetics*, *37*(4–6), 491–512. <https://doi.org/10.1080/02699206.2022.2082887>

Dagmawi, A. G., Hailu, B. H., & Abebe, Y. M. (2023). Effect of Teacher-Mediated Discrete-Trial Training in Improving Communication Skills of Children with Autism Spectrum Disorder. *International Journal of Special Education*, *38*(2). <https://doi.org/10.52291/ijse.2023.38.27>

Daikoku, T., Kumagaya, S., Ayaya, S., & Nagai, Y. (2023). Non-autistic persons modulate their speech rhythm while talking to autistic individuals. *PLOS ONE*, *18*(9), e0285591. <https://doi.org/10.1371/journal.pone.0285591>

Davidson, M. M., & Fleming, K. K. (2023). Story Comprehension Monitoring Across Visual, Listening, and Written Modalities in Children with and Without Autism Spectrum Disorder. *Journal of Autism and Developmental Disorders*, *53*(1), 1–24. <https://doi.org/10.1007/s10803-021-05418-6>

Day, T. C., Malik, I., Boateng, S., Hauschild, K. M., & Lerner, M. D. (2024). Vocal Emotion Recognition in Autism: Behavioral Performance and Event-Related Potential (ERP) Response. *Journal of Autism and Developmental Disorders*, *54*(4), 1235–1248. <https://doi.org/10.1007/s10803-023-05898-8>

De Froy, A., & Rollins, P. R. (2023). The cross-racial/ethnic gesture production of young autistic children and their parents. *Autism & Developmental Language Impairments*, *8*, 23969415231159548. <https://doi.org/10.1177/23969415231159548>

Demopoulos, C., Kopald, B. E., Bangera, N., Paulson, K., & David Lewine, J. (2023). Rapid auditory processing of puretones is associated with basic components of language in individuals with autism spectrum disorders. *Brain and Language*, *238*, 105229. <https://doi.org/10.1016/j.bandl.2023.105229>

Demopoulos, C., Skiba, S. A., Kopald, B. E., Bangera, N., Paulson, K., & Lewine, J. D. (2023). Associations between rapid auditory processing of speech sounds and specific verbal communication skills in autism. *Frontiers in Psychology*, *14*, 1223250. <https://doi.org/10.3389/fpsyg.2023.1223250>

Dindar, K., Loukusa, S., Leinonen, E., Mäkinen, L., Mämmelä, L., Mattila, M.-L., Ebeling, H., & Hurtig, T. (2023). Autistic adults and adults with sub-clinical autistic traits differ from non-autistic adults in social-pragmatic inferencing and narrative discourse. *Autism*, *27*(5), 1320–1335. <https://doi.org/10.1177/13623613221136003>

D’Mello, A. M., Frosch, I. R., Meisler, S. L., Grotzinger, H., Perrachione, T. K., & Gabrieli, J. D. E. (2023). Diminished Repetition Suppression Reveals Selective and Systems-Level Face Processing Differences in ASD. *The Journal of Neuroscience*, *43*(11), 1952–1962. <https://doi.org/10.1523/JNEUROSCI.0608-22.2023>

Doghadze, I., & Gagoshidze, T. (2025). Language phenotypes in children with autism spectrum disorder, expressive language disorder, and typical language development. *Applied Neuropsychology: Child*, *14*(1), 12–22. <https://doi.org/10.1080/21622965.2023.2221359>

Donadio, D. M. D. O., Simões-Zenari, M., Santos, T. H. F., Sanchez, M. G., Molini-Avejonas, D. R., & Cardilli-Dias, D. (2024). Use of the Prompts for Reestructuring Oral Muscular Phonetic Targets (PROMPT) in Autism Spectrum Disorder: A case study. *CoDAS*, *36*(2), e20220299. <https://doi.org/10.1590/2317-1782/20232022299en>

Douglas, S. N., Meadan, H., Biggs, E. E., Bagawan, A., & Terol, A. K. (2023). Building Family Capacity: Supporting multiple family members to implement aided Language modeling. *Journal of Autism and Developmental Disorders*, *53*(7), 2587–2599. <https://doi.org/10.1007/s10803-022-05492-4>

Duan, K., Eyler, L., Pierce, K., Lombardo, M. V., Datko, M., Hagler, D. J., Taluja, V., Zahiri, J., Campbell, K., Barnes, C. C., Arias, S., Nalabolu, S., Troxel, J., Ji, P., & Courchesne, E. (2024). Differences in regional brain structure in toddlers with autism are related to future language outcomes. *Nature Communications*, *15*(1), 5075. <https://doi.org/10.1038/s41467-024-48952-4>

Dunham, K., Zoltowski, A., Feldman, J. I., Davis, S., Rogers, B., Failla, M. D., Wallace, M. T., Cascio, C. J., & Woynaroski, T. G. (2023). Neural Correlates of Audiovisual Speech Processing in Autistic and Non-Autistic Youth. *Multisensory Research*, *36*(3), 263–288. <https://doi.org/10.1163/22134808-bja10093>

Dunham-Carr, K., Feldman, J. I., Simon, D. M., Edmunds, S. R., Tu, A., Kuang, W., Conrad, J. G., Santapuram, P., Wallace, M. T., & Woynaroski, T. G. (2023). The Processing of Audiovisual Speech Is Linked with Vocabulary in Autistic and Nonautistic Children: An ERP Study. *Brain Sciences*, *13*(7), 1043. <https://doi.org/10.3390/brainsci13071043>

Durrleman, S., Bentea, A., Prisecaru, A., Thommen, E., & Delage, H. (2023). Training Syntax to Enhance Theory of Mind in Children with ASD. *Journal of Autism and Developmental Disorders*, *53*(6), 2444–2457. <https://doi.org/10.1007/s10803-022-05507-0>

Duville, M. M., Corona-González, C. E., Romo De León, R., Rodríguez Vera, A., Flores-Jimenez, M. S., Ibarra-Zarate, D. I., & Alonso-Valerdi, L. M. (2023). Perception of task-irrelevant affective prosody by typically developed and diagnosed children with Autism Spectrum Disorder under attentional loads: Electroencephalographic and behavioural data. *Data in Brief*, *48*, 109057. <https://doi.org/10.1016/j.dib.2023.109057>

Edmier, K., Kazee, A., & Yosick, R. (2023). Considerations for practitioners using applied behavior analysis therapy with autistic deaf/hard of hearing clients. *Behavioral Interventions*, *38*(4), 1–12. <https://doi.org/10.1002/bin.1960>

Faerman, A., Sakallah, A., Skiba, S., Kansara, S., Kopald, B. E., Lewine, J. D., & Demopoulos, C. (2023). Language Abilities are Associated with Both Verbal and Nonverbal Intelligence in Children on the Autism Spectrum. *Developmental Neuropsychology*, *48*(5), 248–257. <https://doi.org/10.1080/87565641.2023.2225663>

Farndale, A., & Reichelt, V. (2023). Children’s communicative capital: Promoting inclusive storying in a diverse preschool community through critical participatory action research. *Journal of Early Childhood Literacy*, 14687984231221957. <https://doi.org/10.1177/14687984231221957>

Feldman, J. I., Dunham, K., DiCarlo, G. E., Cassidy, M., Liu, Y., Suzman, E., Williams, Z. J., Pulliam, G., Kaiser, S., Wallace, M. T., & Woynaroski, T. G. (2023). A Randomized Controlled Trial for Audiovisual Multisensory Perception in Autistic Youth. *Journal of Autism and Developmental Disorders*, *53*(11), 4318–4335. <https://doi.org/10.1007/s10803-022-05709-6>

Feldman, J. I., Tu, A., Conrad, J. G., Kuang, W., Santapuram, P., & Woynaroski, T. G. (2023). The Impact of Singing on Visual and Multisensory Speech Perception in Children on the Autism Spectrum. *Multisensory Research*, *36*(1), 57–74. <https://doi.org/10.1163/22134808-bja10087>

Feng, S., Wang, Q., Hu, Y., Lu, H., Li, T., Song, C., Fang, J., Chen, L., & Yi, L. (2023). Increasing audiovisual speech integration in autism through enhanced attention to mouth. *Developmental Science*, *26*(4), e13348. <https://doi.org/10.1111/desc.13348>

Feng, Y., Chen, F., Ma, J., Wang, L., & Peng, G. (2023). Production of Mandarin consonant aspiration and monophthongs in children with Autism Spectrum Disorder. *Clinical Linguistics & Phonetics*, *37*(10), 899–918. <https://doi.org/10.1080/02699206.2022.2099302>

Ferguson, C. J., Preece, D. A., & Schweitzer, R. D. (2023). Alexithymia in autism spectrum disorder. *Australian Psychologist*, *58*(2), 131–137. <https://doi.org/10.1080/00050067.2023.2174409>

Ferguson, J., Dounavi, K., & Craig, E. A. (2023). The Efficacy of Using Telehealth to Coach Parents of Children with Autism Spectrum Disorder on How to Use Naturalistic Teaching to Increase Mands, Tacts and Intraverbals. *Journal of Developmental and Physical Disabilities*, *35*(3), 417–447. <https://doi.org/10.1007/s10882-022-09859-4>

Fittipaldi, S., Armony, J. L., García, A. M., Migeot, J., Cadaveira, M., Ibáñez, A., & Baez, S. (2023). Emotional descriptions increase accidental harm punishment and its cortico-limbic signatures during moral judgment in autism. *Scientific Reports*, *13*(1), 1745. <https://doi.org/10.1038/s41598-023-27709-x>

Fok, M., DeLucia, E. A., Andrzejewski, T., McDonnell, C. G., & Scarpa, A. (2023). Measurement invariance of Child Behavior Checklist internalizing and externalizing factors between non-speaking/partially verbal and speaking autistic children. *Research in Autism Spectrum Disorders*, *108*, 102249. <https://doi.org/10.1016/j.rasd.2023.102249>

Foldager, M., Vestergaard, M., Lassen, J., Petersen, L. S., Oranje, B., Aggernaes, B., & Simonsen, E. (2023). Atypical Semantic Fluency and Recall in Children and Adolescents with Autism Spectrum Disorders Associated with Autism Symptoms and Adaptive Functioning. *Journal of Autism and Developmental Disorders*, *53*(11), 4280–4292. <https://doi.org/10.1007/s10803-022-05677-x>

Foster-Cohen, S., Macrae, T., & Newbury, J. (2023). Variation in morpho-lexical development within and between diagnoses in children with neurodevelopmental disorders. *Frontiers in Psychology*, *13*, 968408. <https://doi.org/10.3389/fpsyg.2022.968408>

Fountain, C., Winter, A. S., Cheslack-Postava, K., & Bearman, P. S. (2023). Developmental Trajectories of Autism. *Pediatrics*, *152*(3), e2022058674. <https://doi.org/10.1542/peds.2022-058674>

Fusaroli, R., Weed, E., Rocca, R., Fein, D., & Naigles, L. (2023a). Caregiver linguistic alignment to autistic and typically developing children: A natural language processing approach illuminates the interactive components of language development. *Cognition*, *236*, 105422. <https://doi.org/10.1016/j.cognition.2023.105422>

Fusaroli, R., Weed, E., Rocca, R., Fein, D., & Naigles, L. (2023b). Repeat After Me? Both Children With and Without Autism Commonly Align Their Language With That of Their Caregivers. *Cognitive Science*, *47*(11), e13369. <https://doi.org/10.1111/cogs.13369>

Gabay, Y., Reinisch, E., Even, D., Binur, N., & Hadad, B.-S. (2024). Intact Utilization of Contextual Information in Speech Categorization in Autism. *Journal of Autism and Developmental Disorders*, *54*(10), 3837–3853. <https://doi.org/10.1007/s10803-023-06106-3>

Garcia, A., Wunderlich, K. L., Pelfrey, C., & Sheppard, C. M. (2024). Experimental analysis of voice volume for children with autism spectrum disorder. *Behavioral Interventions*, *39*(1), e1984. <https://doi.org/10.1002/bin.1984>

Garrido, D., Munoz, J., Fresneda, D., Mendoza, E., Garcia-Retamero, R., & Carballo, G. (2024). Grammatical comprehension in language and communication disorders. *Clinical Linguistics & Phonetics*, *38*(9), 819–837. <https://doi.org/10.1080/02699206.2023.2237647>

Ge, H., Lee, A. K. L., Yuen, H. K., Liu, F., & Yip, V. (2024). Bilingual exposure might enhance L1 development in Cantonese–English bilingual autistic children: Evidence from the production of focus. *Autism*, *28*(7), 1795–1808. <https://doi.org/10.1177/13623613231207449>

Genc-Tosun, D., Kurt, O., Cevher, Z., & Gregori, E. V. (2023). Teaching Children with Autism Spectrum Disorder to Answer Questions Using an iPad-Based Speech-Generating Device. *Journal of Autism and Developmental Disorders*, *53*(9), 3724–3739. <https://doi.org/10.1007/s10803-022-05683-z>

Georgiou, G. P. (2023). Identification of Native Vowels in Normal and Whispered Speech by Individuals with Autism Spectrum Disorder. *Journal of Autism and Developmental Disorders*, *53*(2), 858–862. <https://doi.org/10.1007/s10803-020-04702-1>

Gevarter, C., Prieto, V., Binger, C., & Hartley, M. (2023). Dynamic Assessment of AAC Action Verb Symbols for Children with ASD. *Advances in Neurodevelopmental Disorders*, *7*(3), 329–343. <https://doi.org/10.1007/s41252-022-00312-3>

Gibbs, A. R., Tullis, C. A., Priester, J., & Reddock, C. P. (2024). Teaching Problem Explanations Using Instructive Feedback: A Replication and Extension. *The Analysis of Verbal Behavior*, *40*(2), 248–270. <https://doi.org/10.1007/s40616-023-00195-y>

Gibson, M. T., Schmidt-Kassow, M., & Paulmann, S. (2023). How neurotypical listeners recognize emotions expressed through vocal cues by speakers with high-functioning autism. *PLOS ONE*, *18*(10), e0293233. <https://doi.org/10.1371/journal.pone.0293233>

Gilder, J., & Charlop, M. H. (2023). Increasing Social Communication by Teaching Texting to Autistic Children. *Advances in Neurodevelopmental Disorders*, *7*(3), 403–414. <https://doi.org/10.1007/s41252-023-00322-9>

Gilroy, S. P., McCleery, J. P., & Leader, G. (2023). A delayed intervention start randomized controlled trial of high‐ and low‐tech communication training approaches for school‐age autistic children with co‐occurring intellectual disability. *Journal of Applied Behavior Analysis*, *56*(3), 593–606. <https://doi.org/10.1002/jaba.989>

Gkiolnta, E., Zygopoulou, M., & Syriopoulou-Delli, C. K. (2023). Robot programming for a child with autism spectrum disorder: A pilot study. *International Journal of Developmental Disabilities*, *69*(3), 424–431. <https://doi.org/10.1080/20473869.2023.2194568>

Godel, M., Robain, F., Journal, F., Kojovic, N., Latrèche, K., Dehaene-Lambertz, G., & Schaer, M. (2023). Prosodic signatures of ASD severity and developmental delay in preschoolers. *Npj Digital Medicine*, *6*(1), 99. <https://doi.org/10.1038/s41746-023-00845-4>

Greco, G., Choi, B., Michel, K., & Faja, S. (2023). Here’s the story: Narrative ability and executive function in autism spectrum disorder. *Research in Autism Spectrum Disorders*, *101*, 102092. <https://doi.org/10.1016/j.rasd.2022.102092>

Griffen, B., Holyfield, C., Lorah, E. R., & Caldwell, N. (2024). Increasing linguistic and prelinguistic communication for social closeness during naturalistic AAC instruction with young children on the autism spectrum. *Augmentative and Alternative Communication*, *40*(3), 168–181. <https://doi.org/10.1080/07434618.2023.2283846>

Halbur, M., Kodak, T., Reidy, J., & Bergmann, S. (2024). Comparing Manipulations to Enhance Stimulus Salience during Intraverbal Training. *The Analysis of Verbal Behavior*, *40*(2), 235–247. <https://doi.org/10.1007/s40616-023-00190-3>

Hannigan, L. J., Askeland, R. B., Ask, H., Tesli, M., Corfield, E., Ayorech, Z., Magnus, P., Njølstad, P. R., Øyen, A.-S., Stoltenberg, C., Andreassen, O. A., Ronald, A., Smith, G. D., Reichborn-Kjennerud, T., & Havdahl, A. (2023). Developmental milestones in early childhood and genetic liability to neurodevelopmental disorders. *Psychological Medicine*, *53*(5), 1750–1758. <https://doi.org/10.1017/S0033291721003330>

Hastedt, I., Eisenhower, A., Sheldrick, R. C., & Carter, A. S. (2023). Bilingual and Monolingual Autistic Toddlers: Language and Social Communication Skills. *Journal of Autism and Developmental Disorders*, *53*(6), 2185–2202. <https://doi.org/10.1007/s10803-022-05504-3>

Havdahl, A., Farmer, C., Surén, P., Øyen, A., Magnus, P., Susser, E., Lipkin, W. I., Reichborn‐Kjennerud, T., Stoltenberg, C., Bishop, S., & Thurm, A. (2024). Attainment and loss of early social‐communication skills across neurodevelopmental conditions in the Norwegian Mother, Father and Child Cohort Study. *Journal of Child Psychology and Psychiatry*, *65*(5), 610–619. <https://doi.org/10.1111/jcpp.13792>

Hilviu, D., Frau, F., Bosco, F. M., Marini, A., & Gabbatore, I. (2023). Can Narrative Skills Improve in Autism Spectrum Disorder? A Preliminary Study with Verbally Fluent Adolescents Receiving the Cognitive Pragmatic Treatment. *Journal of Psycholinguistic Research*, *52*(5), 1605–1632. <https://doi.org/10.1007/s10936-023-09945-4>

Hoffmann, J., Travers-Podmaniczky, G., Pelzl, M. A., Brück, C., Jacob, H., Hölz, L., Martinelli, A., & Wildgruber, D. (2023). Impairments in recognition of emotional facial expressions, affective prosody, and multisensory facilitation of response time in high-functioning autism. *Frontiers in Psychiatry*, *14*, 1151665. <https://doi.org/10.3389/fpsyt.2023.1151665>

Hong, Y., Chen, S., Zhou, F., Chan, A., & Tang, T. (2023). Phonetic entrainment in L2 human-robot interaction: An investigation of children with and without autism spectrum disorder. *Frontiers in Psychology*, *14*, 1128976. <https://doi.org/10.3389/fpsyg.2023.1128976>

Horvath, S., & Arunachalam, S. (2023). Assessing receptive verb knowledge in late talkers and autistic children: Advances and cautionary tales. *Journal of Neurodevelopmental Disorders*, *15*(1), 44. <https://doi.org/10.1186/s11689-023-09512-x>

Howard, P. L., & Pagán, A. (2023). No evidence for high inflexible precision of prediction errors in autism during lexical processing. *Autism Research*, *16*(9), 1775–1785. <https://doi.org/10.1002/aur.2994>

Hu, A., Kozloff, V., Owen Van Horne, A., Chugani, D., & Qi, Z. (2024). Dissociation Between Linguistic and Nonlinguistic Statistical Learning in Children with Autism. *Journal of Autism and Developmental Disorders*, *54*(5), 1912–1927. <https://doi.org/10.1007/s10803-023-05902-1>

Hu, X., Lee, G. T., Pan, Q., Gilic, L., & Zeng, S. (2023). Effects of foreign mand training on the emergence of foreign tact and listener responses for Chinese‐speaking children with autism spectrum disorder. *Behavioral Interventions*, *38*(2), 437–455. <https://doi.org/10.1002/bin.1930>

Hudry, K., Smith, J., Pillar, S., Varcin, K. J., Bent, C. A., Boutrus, M., Chetcuti, L., Clark, A., Dissanayake, C., Iacono, T., Kennedy, L., Lant, A., Robinson Lake, J., Segal, L., Slonims, V., Taylor, C., Wan, M. W., Green, J., & Whitehouse, A. J. O. (2023). The Utility of Natural Language Samples for Assessing Communication and Language in Infants Referred with Early Signs of Autism. *Research on Child and Adolescent Psychopathology*, *51*(4), 529–539. <https://doi.org/10.1007/s10802-022-01010-3>

Huettig, F., Voeten, C. C., Pascual, E., Liang, J., & Hintz, F. (2023). Do autistic children differ in language-mediated prediction? *Cognition*, *239*, 105571. <https://doi.org/10.1016/j.cognition.2023.105571>

Iao, L.-S., Shen, C.-W., & Wu, C.-C. (2024). A Longitudinal Study of Joint Attention, Motor Imitation and Language Development in Young Children with Autism Spectrum Disorder in Taiwan. *Journal of Autism and Developmental Disorders*, *54*(7), 2651–2662. <https://doi.org/10.1007/s10803-023-05950-7>

Irwin, J., Harwood, V., Kleinman, D., Baron, A., Avery, T., Turcios, J., & Landi, N. (2023). Neural and Behavioral Differences in Speech Perception for Children With Autism Spectrum Disorders Within an Audiovisual Context. *Journal of Speech, Language, and Hearing Research*, *66*(7), 2390–2403. <https://doi.org/10.1044/2023_JSLHR-22-00661>

Issa, H. M. B., Ying, J. H., & Azam, Y. B. (2023). Comprehension of wh-questions among Jordanian children with autism spectrum disorder and specific language impairment. *Humanities and Social Sciences Communications*, *10*(1), 601. <https://doi.org/10.1057/s41599-023-02118-7>

Jasmin, K., Martin, A., & Gotts, S. J. (2023). Atypical connectivity aids conversation in autism. *Scientific Reports*, *13*(1), 5303. <https://doi.org/10.1038/s41598-023-32249-5>

Joginder Singh, S., & Loo, Z. L. (2023). The use of augmentative and alternative communication by children with developmental disability in the classroom: A case study. *Disability and Rehabilitation: Assistive Technology*, *18*(8), 1281–1289. <https://doi.org/10.1080/17483107.2023.2196305>

Jones, M. K., Sone, B. J., Grauzer, J., Sudec, L., Kaat, A., & Roberts, M. Y. (2024). Characterizing mechanisms of caregiver-mediated naturalistic developmental behavioral interventions for autistic toddlers: A randomized clinical trial. *Autism*, *28*(7), 1847–1860. <https://doi.org/10.1177/13623613231213283>

Kasari, C., Shire, S., Shih, W., Landa, R., Levato, L., & Smith, T. (2023). Spoken language outcomes in limited language preschoolers with autism and global developmental delay: RCT of early intervention approaches. *Autism Research*, *16*(6), 1236–1246. <https://doi.org/10.1002/aur.2932>

Ke, Y., & Zhou, X. (2024). Perceptive assessment for metaphoric disability in autistic children: An inhibition-of-return approach. *Current Psychology*, *43*(1), 51–61. <https://doi.org/10.1007/s12144-023-05324-3>

Kedar, M., & Bauminger‐Zviely, N. (2023). Predictors of individual differences in minimally verbal peer communication exchanges following peer‐oriented social intervention. *Autism Research*, *16*(1), 230–244. <https://doi.org/10.1002/aur.2852>

Khorasani, M., Kahani, M., Yazdi, S. A. A., & Hajiaghaei-Keshteli, M. (2023). Towards finding the lost generation of autistic adults: A deep and multi-view learning approach on social media. *Knowledge-Based Systems*, *276*, 110724. <https://doi.org/10.1016/j.knosys.2023.110724>

Kim, S., Cha, J., Kim, D., & Park, E. (2023). Understanding Mental Health Issues in Different Subdomains of Social Networking Services: Computational Analysis of Text-Based Reddit Posts. *Journal of Medical Internet Research*, *25*, e49074. <https://doi.org/10.2196/49074>

Kimhi, Y., Mishkin, I. K., & Bauminger-Zviely, N. (2023). Reading comprehension strategies for expository texts: Children with and without ASD. *Research in Autism Spectrum Disorders*, *105*, 102169. <https://doi.org/10.1016/j.rasd.2023.102169>

Kishel, C., & Vollmer, T. (2023). An Assessment of Response to Conversation Cues of Uninterest Conducted via Telehealth. *Behavior Modification*, *47*(2), 454–475. <https://doi.org/10.1177/01454455221142025>

Kılıç-Tülü, B., Ökcün-Akçamuş, M. Ç., & Ergül, C. (2023). Investigation of Early Literacy Skills in Children on the Autism Spectrum: The Case of Turkish-Speaking Children. *Journal of Autism and Developmental Disorders*, *53*(6), 2395–2408. <https://doi.org/10.1007/s10803-022-05456-8>

Kohn, B. H., Vidal, P., Chiao, R., Pantalone, D. W., & Faja, S. (2023). Sexual Knowledge, Experiences, and Pragmatic Language in Adults With and Without Autism: Implications for Sex Education. *Journal of Autism and Developmental Disorders*, *53*(10), 3770–3786. <https://doi.org/10.1007/s10803-022-05659-z>

Koudys, J., Perry, A., Magnacca, C., & McFee, K. (2023). Predictors of Picture Exchange Communication System (PECS) outcomes. *Autism & Developmental Language Impairments*, *8*, 23969415231221516. <https://doi.org/10.1177/23969415231221516>

Laçin, E. (2024). Increasing Vocabulary and Listening Comprehension During Adapted Shared Reading: An Intervention for Preschoolers with Autism Spectrum Disorder. *Journal of Autism and Developmental Disorders*, *54*(12), 4376–4393. <https://doi.org/10.1007/s10803-023-06149-6>

Lau, J. C. Y., Losh, M., & Speights, M. (2023). Differences in speech articulatory timing and associations with pragmatic language ability in autism. *Research in Autism Spectrum Disorders*, *102*, 102118. <https://doi.org/10.1016/j.rasd.2023.102118>

Laubscher, E., Pope, L., & Light, J. (2024). “You Just Want to Be Able to Communicate With Your Child”: Parents’ Perspectives on Communication and AAC Use for Beginning Communicators on the Autism Spectrum. *American Journal of Speech-Language Pathology*, *33*(2), 716–735. <https://doi.org/10.1044/2023_AJSLP-23-00254>

Leipold, S., Abrams, D. A., Karraker, S., Phillips, J. M., & Menon, V. (2023). Aberrant Emotional Prosody Circuitry Predicts Social Communication Impairments in Children With Autism. *Biological Psychiatry: Cognitive Neuroscience and Neuroimaging*, *8*(5), 531–541. <https://doi.org/10.1016/j.bpsc.2022.09.016>

Leung, F. Y. N., Stojanovik, V., Micai, M., Jiang, C., & Liu, F. (2023). Emotion recognition in autism spectrum disorder across age groups: A cross‐sectional investigation of various visual and auditory communicative domains. *Autism Research*, *16*(4), 783–801. <https://doi.org/10.1002/aur.2896>

Li, C., & Hu, J. (2024). Relatively Inefficient Integration of Metaphorical Semantics in Autistic Adults Without Intellectual Impairment. *Journal of Autism and Developmental Disorders*, *54*(6), 2254–2265. <https://doi.org/10.1007/s10803-023-05964-1>

Li, L., Su, Y. (Esther), Hou, W., Zhou, M., Xie, Y., Zou, X., & Li, M. (2023). Expressive Language Profiles in a Clinical Screening Sample of Mandarin-Speaking Preschool Children With Autism Spectrum Disorder. *Journal of Speech, Language, and Hearing Research*, *66*(11), 4497–4518. <https://doi.org/10.1044/2023_JSLHR-23-00184>

Li, X., Peng, Y., Lu, Y., & Zhang, Y. (2024). The effect of recasting by mothers with different conversational styles on the communication behavior of autistic children: Lag sequential analysis. *Autism Research*, *17*(1), 125–137. <https://doi.org/10.1002/aur.3052>

Liu, J., Wang, Y., & Yi, L. (2023). Heterogeneity and imbalance of reading profiles in Autism Spectrum Disorders. *Research in Autism Spectrum Disorders*, *100*, 102088. <https://doi.org/10.1016/j.rasd.2022.102088>

Liu, M., Brady, N. C., Boorom, O., Fleming, K., Yue, J., & Liu, Q. (2024). Prelinguistic communication complexity predicts expressive language in initial minimally verbal autistic children. *International Journal of Language & Communication Disorders*, *59*(1), 413–425. <https://doi.org/10.1111/1460-6984.12956>

Logan, K., Iacono, T., & Trembath, D. (2024). Aided Enhanced milieu teaching to develop symbolic and social communication skills in children with autism spectrum disorder. *Augmentative and Alternative Communication*, *40*(2), 125–139. <https://doi.org/10.1080/07434618.2023.2263558>

Luo, L., & Su, I.-F. (2023). Meta-linguistic awareness skills in Chinese-speaking children with hyperlexia: A single-case study. *Frontiers in Psychology*, *14*, 1049775. <https://doi.org/10.3389/fpsyg.2023.1049775>

MacDonald-Prégent, A., Saiyed, F., Hyde, K., Sharda, M., & Nadig, A. (2024). Response to Music-Mediated Intervention in Autistic Children with Limited Spoken Language Ability. *Journal of Autism and Developmental Disorders*, *54*(4), 1438–1452. <https://doi.org/10.1007/s10803-022-05872-w>

Maes, P., Weyland, M., & Kissine, M. (2023). Describing (pre)linguistic oral productions in 3- to 5-year-old autistic children: A cluster analysis. *Autism*, *27*(4), 967–982. <https://doi.org/10.1177/13623613221122663>

Maes, P., Weyland, M., & Kissine, M. (2024). Structure and acoustics of the speech of verbal autistic preschoolers. *Journal of Child Language*, *51*(3), 509–525. <https://doi.org/10.1017/S0305000923000417>

Maksimović, S., Marisavljević, M., Stanojević, N., Ćirović, M., Punišić, S., Adamović, T., Đorđević, J., Krgović, I., & Subotić, M. (2023). Importance of Early Intervention in Reducing Autistic Symptoms and Speech–Language Deficits in Children with Autism Spectrum Disorder. *Children*, *10*(1), 122. <https://doi.org/10.3390/children10010122>

Malmir, Z., & Soltani, N. (2023). The Effect of Reciprocal Imitation Training on Increasing Expressive Vocabulary of Children with Autism Spectrum Disorders. *Romanian Journal of Military Medicine*, *126*(1), 40–47. <https://doi.org/10.55453/rjmm.2023.126.1.7>

Maltman, N., Hilvert, E., Friedman, L., & Sterling, A. (2023). Comparison of Linguistic Error Production in Conversational Language Among Boys With Fragile X Syndrome + Autism Spectrum Disorder and Autistic Boys. *Journal of Speech, Language, and Hearing Research*, *66*(1), 296–313. <https://doi.org/10.1044/2022_JSLHR-22-00078>

Maltman, N., Willer, R., & Sterling, A. (2023). An Exploratory Study of Pragmatic Language Use Across Contexts With the Pragmatic Rating Scale–School Age Among Autistic Boys and Boys With Fragile X Syndrome Plus Autism. *Journal of Speech, Language, and Hearing Research*, *66*(11), 4547–4557. <https://doi.org/10.1044/2023_JSLHR-23-00024>

Manenti, M., Tuller, L., Houy-Durand, E., Bonnet-Brilhault, F., & Prévost, P. (2023). Assessing structural language skills of autistic adults: Focus on sentence repetition. *Lingua*, *294*, 103598. <https://doi.org/10.1016/j.lingua.2023.103598>

Mantzoros, T., Lee, D. L., Ajemigbitse, A. R., & Stover, L. J. (2023). Implementing a differential reinforcement of low rates schedule to alter vocal stereotypy and task engagement in two adolescents with autism spectrum disorder. *Behavioral Interventions*, *38*(3), 689–705. <https://doi.org/10.1002/bin.1948>

Marom, M. K., Gilboa, A., & Bodner, E. (2024). Conversations on echolalia: A qualitative inquiry into autistic adults’ views on echolalia, language, and music. *Nordic Journal of Music Therapy*, *33*(3), 169–188. <https://doi.org/10.1080/08098131.2023.2268690>

Márquez-García, A. V., Ng, B. K., Iarocci, G., Moreno, S., Vakorin, V. A., & Doesburg, S. M. (2023). Atypical Associations between Functional Connectivity during Pragmatic and Semantic Language Processing and Cognitive Abilities in Children with Autism. *Brain Sciences*, *13*(10), 1448. <https://doi.org/10.3390/brainsci13101448>

Martin, G. E., Lee, M., Bicknell, K., Goodkind, A., Maltman, N., & Losh, M. (2023). A longitudinal investigation of pragmatic language across contexts in autism and related neurodevelopmental conditions. *Frontiers in Neurology*, *14*, 1155691. <https://doi.org/10.3389/fneur.2023.1155691>

McNair, M. L., Keenan, E. G., Houck, A. P., & Lerner, M. D. (2024). Seeking contexts that promote neurodiverse social success: Patterns of behavior during minimally-structured interaction settings in autistic and non-autistic youth. *Development and Psychopathology*, *36*(4), 1669–1684. <https://doi.org/10.1017/S0954579423000950>

Michel, L., Ricou, C., Bonnet-Brilhault, F., Houy-Durand, E., & Latinus, M. (2024). Sounds Pleasantness Ratings in Autism: Interaction Between Social Information and Acoustical Noise Level. *Journal of Autism and Developmental Disorders*, *54*(6), 2148–2157. <https://doi.org/10.1007/s10803-023-05989-6>

Minnigulova, A., Davydova, E., Pereverzeva, D., Sorokin, A., Tyushkevich, S., Mamokhina, U., Danilina, K., Dragoy, O., & Arutiunian, V. (2023). Corpus callosum organization and its implication to core and co-occurring symptoms of Autism Spectrum Disorder. *Brain Structure and Function*, *228*(3–4), 775–785. <https://doi.org/10.1007/s00429-023-02617-y>

Miranda, A., Berenguer, C., Baixauli, I., & Roselló, B. (2023). Childhood language skills as predictors of social, adaptive and behavior outcomes of adolescents with autism spectrum disorder. *Research in Autism Spectrum Disorders*, *103*, 102143. <https://doi.org/10.1016/j.rasd.2023.102143>

Muldoon, D. M., & Gray, R. (2023). Teaching Receptive Vocabulary to Minimally Verbal Preschoolers With Autism Spectrum Disorder: A Single-Case Multiple Baseline Design. *American Journal of Speech-Language Pathology*, *32*(6), 3036–3047. <https://doi.org/10.1044/2023_AJSLP-23-00095>

Muller, K. F., Paula Nunes, D. R., & Schmidt, C. (2023). Caregiver Implemented Dialogic Reading: Impact on children with and without autism in Brazil. *International Journal of Special Education (IJSE)*, *38*(1), 47–57. <https://doi.org/10.52291/ijse.2023.38.5>

Naples, A., Tenenbaum, E. J., Jones, R. N., Righi, G., Sheinkopf, S. J., & Eigsti, I.-M. (2023). Exploring communicative competence in autistic children who are minimally verbal: The Low Verbal Investigatory Survey for Autism (LVIS). *Autism*, *27*(5), 1391–1406. <https://doi.org/10.1177/13623613221136657>

O’Brien, A. M., Perrachione, T. K., Wisman Weil, L., Sanchez Araujo, Y., Halverson, K., Harris, A., Ostrovskaya, I., Kjelgaard, M., Kenneth Wexler, Tager-Flusberg, H., Gabrieli, J. D. E., & Qi, Z. (2023). Altered engagement of the speech motor network is associated with reduced phonological working memory in autism. *NeuroImage: Clinical*, *37*, 103299. <https://doi.org/10.1016/j.nicl.2022.103299>

Ochi, K., Kojima, M., Ono, N., Kuroda, M., Owada, K., Sagayama, S., & Yamasue, H. (2024). Objective assessment of autism spectrum disorder based on performance in structured interpersonal acting‐out tasks with prosodic stability and variability. *Autism Research*, *17*(2), 395–409. <https://doi.org/10.1002/aur.3080>

Oosting, D. R., Howard, M. S., & Carter, A. S. (2024). Reciprocal Associations Between Language Ability and Social Functioning Development in Pre-verbal Autistic Children. *Journal of Autism and Developmental Disorders*, *54*(5), 1643–1655. <https://doi.org/10.1007/s10803-023-05906-x>

Orozco, D., Cividini-Motta, C., Campos, C., & Brolmann, H. A. (2023). Teaching Mands: Correspondence among Acquisition, Recommendations of the Essential for Living Communication Modality Assessment, and Preference. *Behavior Analysis in Practice*, *16*(3), 812–825. <https://doi.org/10.1007/s40617-022-00764-y>

O’Shea, A., Holmes, C. H. B., & Engelhardt, P. E. (2023). A Parental-Report Questionnaire for Language Abilities and Pragmatics in Children and Adolescents with Autism Spectrum Disorders. *Brain Sciences*, *13*(2), 196. <https://doi.org/10.3390/brainsci13020196>

Osos, J. A., Higbee, T. S., A. Lindgren, N., & Campbell, V. E. (2024). The Use of Matrix Training to Teach Color–Shape Tacts Through Telehealth. *The Analysis of Verbal Behavior*, *40*(2), 118–134. <https://doi.org/10.1007/s40616-023-00193-0>

Ostrolenk, A., Courchesne, V., & Mottron, L. (2023). A longitudinal study on language acquisition in monozygotic twins concordant for autism and hyperlexia. *Brain and Cognition*, *173*, 106099. <https://doi.org/10.1016/j.bandc.2023.106099>

O’Sullivan, J., Bogaarts, G., Schoenenberger, P., Tillmann, J., Slater, D., Mesgarani, N., Eule, E., Kilchenmann, T., Murtagh, L., Hipp, J., Lindemann, M., Lipsmeier, F., Cheng, W.-Y., Nobbs, D., & Chatham, C. (2023). Automatic speaker diarization for natural conversation analysis in autism clinical trials. *Scientific Reports*, *13*(1), 10270. <https://doi.org/10.1038/s41598-023-36701-4>

Owen, T. M., & Rodriguez, N. M. (2024). Toward establishing a qualifying autoclitic repertoire in children with autism spectrum disorder. *Journal of Applied Behavior Analysis*, *57*(1), 204–225. <https://doi.org/10.1002/jaba.1026>

Patel, S. P., Landau, E., Martin, G. E., Rayburn, C., Elahi, S., Fragnito, G., & Losh, M. (2023). A profile of prosodic speech differences in individuals with autism spectrum disorder and first-degree relatives. *Journal of Communication Disorders*, *102*, 106313. <https://doi.org/10.1016/j.jcomdis.2023.106313>

Paynter, J., O’Leary, K., & Westerveld, M. (2024). Pre-school Skills and School-Age Reading Comprehension in Children on the Autism Spectrum: A Preliminary Investigation. *Journal of Autism and Developmental Disorders*, *54*(5), 1834–1848. <https://doi.org/10.1007/s10803-023-05949-0>

Peeters, R., Premchand, A., & Tops, W. (2025). Neuropsychological profile of children with Autism Spectrum Disorder and children with Developmental Language Disorder and its relationship with social communication. *Applied Neuropsychology: Child*, *14*(1), 1–11. <https://doi.org/10.1080/21622965.2023.2211703>

Pelzl, M. A., Travers-Podmaniczky, G., Brück, C., Jacob, H., Hoffmann, J., Martinelli, A., Hölz, L., Wabersich-Flad, D., & Wildgruber, D. (2023). Reduced impact of nonverbal cues during integration of verbal and nonverbal emotional information in adults with high-functioning autism. *Frontiers in Psychiatry*, *13*, 1069028. <https://doi.org/10.3389/fpsyt.2022.1069028>

Peristeri, E., Kamona, X., & Varlokosta, S. (2024). The Acquisition of Relative Clauses in Autism: The Role of Executive Functions and Language. *Journal of Autism and Developmental Disorders*, *54*(12), 4394–4407. <https://doi.org/10.1007/s10803-023-06159-4>

Pham, L. N. H., Lee, A. K., Estes, A., Dager, S., Hemingway, S. J. (Astley), Thorne, J. C., & Lau, B. K. (2024). Comparing narrative storytelling ability in individuals with autism and fetal alcohol spectrum disorders. *International Journal of Language & Communication Disorders*, *59*(2), 779–797. <https://doi.org/10.1111/1460-6984.12964>

Phan, L., Tariq, A., Lam, G., Mirza, M., Paiva, D., Lazic, M., Emami, Z., Anagnostou, E., Gordon, K. A., & Pang, E. W. (2023). Children with autism spectrum disorder who demonstrate normal language scores use a bottom‐up semantic processing strategy: Evidence from N400 recordings. *Brain and Behavior*, *13*(9), e3158. <https://doi.org/10.1002/brb3.3158>

Pierce, K., Wen, T. H., Zahiri, J., Andreason, C., Courchesne, E., Barnes, C. C., Lopez, L., Arias, S. J., Esquivel, A., & Cheng, A. (2023). Level of Attention to Motherese Speech as an Early Marker of Autism Spectrum Disorder. *JAMA Network Open*, *6*(2), e2255125. <https://doi.org/10.1001/jamanetworkopen.2022.55125>

Piltz, V. J., Halldner, L., Markus, J.-F., Fridell, A., Bölte, S., & Choque Olsson, N. (2024). Symptom similarities and differences in social interaction between autistic children and adolescents with and without ADHD. *Current Psychology*, *43*(4), 3503–3513. <https://doi.org/10.1007/s12144-023-04499-z>

Pirinen, V., Loukusa, S., Dindar, K., Mäkinen, L., Hurtig, T., Jussila, K., Mattila, M.-L., & Eggers, K. (2023). A Comprehensive Analysis of Speech Disfluencies in Autistic Young Adults and Control Young Adults: Group Differences in Typical, Stuttering-Like, and Atypical Disfluencies. *Journal of Speech, Language, and Hearing Research*, *66*(3), 832–848. <https://doi.org/10.1044/2022_JSLHR-22-00265>

Plank, I. S., Koehler, J. C., Nelson, A. M., Koutsouleris, N., & Falter-Wagner, C. M. (2023). Automated extraction of speech and turn-taking parameters in autism allows for diagnostic classification using a multivariable prediction model. *Frontiers in Psychiatry*, *14*, 1257569. <https://doi.org/10.3389/fpsyt.2023.1257569>

Poole, D., Gowen, E., Poliakoff, E., Lambrechts, A., & Jones, L. A. (2024). When 2 become 1: Autistic simultaneity judgements about asynchronous audiovisual speech. *Quarterly Journal of Experimental Psychology*, *77*(9), 1865–1882. <https://doi.org/10.1177/17470218231197518>

Ré, T. C., Rieken, C. J., Brandt, J. A., Pacitto, G. O., & Yepez, J. (2024). Differential Reinforcement of Low Frequency Behavior as an Interdependent Group Contingency for Children Diagnosed with Autism Spectrum Disorder. *Journal of Behavioral Education*, *33*(4), 949–964. <https://doi.org/10.1007/s10864-023-09512-w>

Reed, P. (2023). Individuals with autism spectrum disorder are differentially sensitive to interference from previous verbal feedback. *Autism*, *27*(7), 2011–2020. <https://doi.org/10.1177/13623613221150377>

Reindal, L., Nærland, T., Weidle, B., Lydersen, S., Andreassen, O. A., & Sund, A. M. (2023). Structural and Pragmatic Language Impairments in Children Evaluated for Autism Spectrum Disorder (ASD). *Journal of Autism and Developmental Disorders*, *53*(2), 701–719. <https://doi.org/10.1007/s10803-020-04853-1>

Reyes, N., Soke, G. N., Wiggins, L., Barger, B., Moody, E., Rosenberg, C., Schieve, L., Reaven, J., Reynolds, A. M., & Hepburn, S. (2024). Social and language regression: Characteristics of children with autism spectrum disorder in a community-based sample. *Journal of Developmental and Physical Disabilities*, *36*(4), 713–728. <https://doi.org/10.1007/s10882-023-09929-1>

Rimmer, C., Dahary, H., & Quintin, E.-M. (2024). Links between musical beat perception and phonological skills for autistic children. *Child Neuropsychology*, *30*(3), 361–380. <https://doi.org/10.1080/09297049.2023.2202902>

Rimmer, C., Philibert-Lignières, G., Iarocci, G., & Quintin, E.-M. (2024). The Contribution of Perceptual Reasoning Skills to Phonological Awareness for School Age Autistic Children. *Journal of Autism and Developmental Disorders*, *54*(4), 1361–1375. <https://doi.org/10.1007/s10803-022-05834-2>

Roberts, M. Y., Stern, Y. S., Grauzer, J., Nietfeld, J., Thompson, S., Jones, M., Kaat, A. J., & Kaiser, A. P. (2023). Teaching Caregivers to Support Social Communication: Results From a Randomized Clinical Trial of Autistic Toddlers. *American Journal of Speech-Language Pathology*, *32*(1), 115–127. <https://doi.org/10.1044/2022_AJSLP-22-00133>

Rong, Y. (2024). Comprehension of Spatial Demonstratives in Mandarin-speaking Children on the Autism Spectrum: The Roles of Theory of Mind and Executive Function. *Journal of Autism and Developmental Disorders*, *54*(11), 4288–4301. <https://doi.org/10.1007/s10803-023-06111-6>

Rong, Y., Weng, Y., Chen, F., & Peng, G. (2023). Categorical perception of Mandarin lexical tones in language-delayed autistic children. *Autism*, *27*(5), 1426–1437. <https://doi.org/10.1177/13623613221138687>

Rothwell, C., Westermann, G., & Hartley, C. (2024). How do Autistic and Neurotypical Children’s Interests Influence their Accuracy During Novel Word Learning? *Journal of Autism and Developmental Disorders*, *54*(9), 3301–3315. <https://doi.org/10.1007/s10803-023-06066-8>

Ruiz Callejo, D., Wouters, J., & Boets, B. (2023). Speech‐in‐noise perception in autistic adolescents with and without early language delay. *Autism Research*, *16*(9), 1719–1727. <https://doi.org/10.1002/aur.2966>

Ryan, C., & Cogan, S. (2023). Eliciting Expressions of Emotion: An Exploratory Analysis of Alexithymia in Adults with Autism Utilising the APRQ. *Journal of Autism and Developmental Disorders*, *53*(6), 2499–2513. <https://doi.org/10.1007/s10803-022-05508-z>

Safi, M. F., Al Sadrani, B., & Mustafa, A. (2023). Virtual voice assistant applications improved expressive verbal abilities and social interactions in children with autism spectrum disorder: A Single-Subject experimental study. *International Journal of Developmental Disabilities*, *69*(4), 555–567. <https://doi.org/10.1080/20473869.2021.1977596>

Sakin, B., Kaftar, Y., & Albay, G. (2023). The Effect of Autism Spectrum Disorder on the Processing of Neural Response Metaphors. *Clinical and Experimental Health Sciences*, *13*(1), 228–233. <https://doi.org/10.33808/clinexphealthsci.1039708>

Sawchak, A., Waddington, H., & Sigafoos, J. (2023). Teaching Multi-step Requesting and Social Communication to Five Autistic Children Using Speech-Generating Devices and Systematic Instruction. *Advances in Neurodevelopmental Disorders*, *7*(3), 344–352. <https://doi.org/10.1007/s41252-023-00320-x>

Schelinski, S., & Von Kriegstein, K. (2023). Responses in left inferior frontal gyrus are altered for speech‐in‐noise processing, but not for clear speech in autism. *Brain and Behavior*, *13*(2), e2848. <https://doi.org/10.1002/brb3.2848>

Schroeder, K., Rosselló, J., Torrades, T. R., & Hinzen, W. (2023). Linguistic markers of autism spectrum conditions in narratives: A comprehensive analysis. *Autism & Developmental Language Impairments*, *8*, 23969415231168557. <https://doi.org/10.1177/23969415231168557>

Schwartzberg, E. T., & Silverman, M. J. (2023). Comparing the comprehension of short stories paired with familiar and unfamiliar melodies and spoken text in autistic children. *Psychology of Music*, *51*(6), 1571–1583. <https://doi.org/10.1177/03057356231153058>

Shevchuk-Hill, S., Szczupakiewicz, S., Kofner, B., & Gillespie-Lynch, K. (2023). Comparing narrative writing of autistic and non-autistic College students. *Journal of Autism and Developmental Disorders*, *53*(10), 3901–3915. <https://doi.org/10.1007/s10803-022-05516-z>

Shillingsburg, M. A., Bartlett, B., Thompson, T., McCracken, C., & Scahill, L. (2024). A Feasibility Trial of Response Contingent Stimulus-Stimulus Pairing to Promote Vocalizations in Minimally Verbal Children Diagnosed with Autism. *Journal of Developmental and Physical Disabilities*, *36*(2), 391–408. <https://doi.org/10.1007/s10882-023-09913-9>

Smith, J., Chetcuti, L., Kennedy, L., Varcin, K. J., Slonims, V., Bent, C. A., Green, J., Iacono, T., Pillar, S., Taylor, C., Wan, M. W., Whitehouse, A. J. O., Hudry, K., & the AICES Team. (2023). Caregiver sensitivity predicts infant language use, and infant language complexity predicts caregiver language complexity, in the context of possible emerging autism. *Autism Research*, *16*(4), 745–756. <https://doi.org/10.1002/aur.2879>

Smith, J., Sulek, R., Van Der Wert, K., Cincotta-Lee, O., Green, C. C., Bent, C. A., Chetcuti, L., & Hudry, K. (2023). Parental Imitations and Expansions of Child Language Predict Later Language Outcomes of Autistic Preschoolers. *Journal of Autism and Developmental Disorders*, *53*(11), 4107–4120. <https://doi.org/10.1007/s10803-022-05706-9>

Solis, M., & McKenna, J. W. (2025). Reading Instruction for Students with Autism Spectrum Disorder: Comparing Observations of Instruction to Student Reading Profiles. *Journal of Behavioral Education*, *34*(2), 399–419. <https://doi.org/10.1007/s10864-023-09532-6>

Soltiyeva, A., Oliveira, W., Madina, A., Adilkhan, S., Urmanov, M., & Hamari, J. (2023). My Lovely Granny’s Farm: An immersive virtual reality training system for children with autism spectrum disorder. *Education and Information Technologies*, *28*(12), 16887–16907. <https://doi.org/10.1007/s10639-023-11862-x>

Song, Y., Nie, Z., & Shan, J. (2024). Comprehension of irony in autistic children: The role of theory of mind and executive function. *Autism Research*, *17*(1), 109–124. <https://doi.org/10.1002/aur.3051>

Spinks, H. M., Falcomata, T. S., & Spinks, E. M. (2023). An evaluation of a multi‐component intervention for loud speech among children with autism spectrum disorder. *Behavioral Interventions*, *38*(2), 512–523. <https://doi.org/10.1002/bin.1931>

Sterrett, K., Holbrook, A., Landa, R., Kaiser, A., & Kasari, C. (2023). The effect of responsiveness to speech-generating device input on spoken language in children with autism spectrum disorder who are minimally verbal^†^. *Augmentative and Alternative Communication*, *39*(1), 23–32. <https://doi.org/10.1080/07434618.2022.2120070>

Strohmeier, C. W., Goetzel, A., Deinlein, S., & Schmidt, J. D. (2023). Repetitive Speech and Problem Behavior: Functional Analysis of Precurrent Contingencies. *Behavior Analysis in Practice*, *16*(3), 745–754. <https://doi.org/10.1007/s40617-022-00743-3>

Sturrock, A., Foy, K., Freed, J., Adams, C., & Leadbitter, K. (2023). The impact of subtle language and communication difficulties on the daily lives of autistic children without intellectual disability: Parent perspectives. *International Journal of Language & Communication Disorders*, *58*(4), 1232–1250. <https://doi.org/10.1111/1460-6984.12859>

Su, W.-C., Culotta, M., Mueller, J., Tsuzuki, D., & Bhat, A. (2023a). fNIRS-Based Differences in Cortical Activation during Tool Use, Pantomimed Actions, and Meaningless Actions between Children with and without Autism Spectrum Disorder (ASD). *Brain Sciences*, *13*(6), 876. <https://doi.org/10.3390/brainsci13060876>

Su, W.-C., Culotta, M., Mueller, J., Tsuzuki, D., & Bhat, A. N. (2023b). Autism-Related Differences in Cortical Activation When Observing, Producing, and Imitating Communicative Gestures: An fNIRS Study. *Brain Sciences*, *13*(9), 1284. <https://doi.org/10.3390/brainsci13091284>

Suhr, M., Bean, A., Rolniak, J., Paden Cargill, L., & Lyle, S. (2024). The influence of classroom context on AAC device use for nonspeaking school-aged autistic children. *International Journal of Speech-Language Pathology*, *26*(3), 434–444. <https://doi.org/10.1080/17549507.2023.2220992>

Sukenik, N. (2025). Relative clause production abilities of Hebrew-speaking children with ASD. *Language Acquisition*, *32*(1), 1–22. <https://doi.org/10.1080/10489223.2023.2197888>

Taddei, M., Bulgheroni, S., Toffalini, E., Pantaleoni, C., & Lanfranchi, S. (2023). Developmental profiles of young children with autism spectrum disorder and global developmental delay: A study with the Griffiths III scales. *Autism Research*, *16*(7), 1344–1359. <https://doi.org/10.1002/aur.2953>

Tang, B., Levine, M., Adamek, J. H., Wodka, E. L., Caffo, B. S., & Ewen, J. B. (2023). Evaluating causal psychological models: A study of language theories of autism using a large sample. *Frontiers in Psychology*, *14*, 1060525. <https://doi.org/10.3389/fpsyg.2023.1060525>

Tantucci, V., & Wang, A. (2023). Dialogic Priming and Dynamic Resonance in Autism: Creativity Competing with Engagement in Chinese Children with ASD. *Journal of Autism and Developmental Disorders*, *53*(6), 2458–2474. <https://doi.org/10.1007/s10803-022-05505-2>

Teh, E. J., & Yap, M. J. (2023). Short report: Social processing in non-emotional contexts by children with and without autism spectrum disorders (ASD). *PLOS ONE*, *18*(5), e0285972. <https://doi.org/10.1371/journal.pone.0285972>

Teimouri Sangani, M., Ansari, N. N., Soleymani, Z., Jalilevand, N., Sohrabi, M., Mohamadi, R., & Razjouyan, K. (2023). Narrative Microstructure and Macrostructure Skills of Persian-Speaking Children with Autism Spectrum Disorder. *Medical Journal of The Islamic Republic of Iran*. <https://doi.org/10.47176/mjiri.37.119>

Thomas, R. P., Wittke, K., Blume, J., Mastergeorge, A. M., & Naigles, L. (2023). Predicting Language in Children with ASD Using Spontaneous Language Samples and Standardized Measures. *Journal of Autism and Developmental Disorders*, *53*(10), 3916–3931. <https://doi.org/10.1007/s10803-022-05691-z>

Thompson, E., Feldman, J. I., Valle, A., Davis, H., Keceli-Kaysili, B., Dunham, K., Woynaroski, T., Tharpe, A. M., & Picou, E. M. (2023). A Comparison of Listening Skills of Autistic and Non-Autistic Youth While Using and Not Using Remote Microphone Systems. *Journal of Speech, Language, and Hearing Research*, *66*(11), 4618–4634. <https://doi.org/10.1044/2023_JSLHR-22-00720>

Todd, J. T., & Bahrick, L. E. (2023). Individual Differences in Multisensory Attention Skills in Children with Autism Spectrum Disorder Predict Language and Symptom Severity: Evidence from the Multisensory Attention Assessment Protocol (MAAP). *Journal of Autism and Developmental Disorders*, *53*(12), 4685–4710. <https://doi.org/10.1007/s10803-022-05752-3>

Tofani, M., Scarcella, L., Galeoto, G., Giovannone, F., & Sogos, C. (2023). Behavioral gender differences across Pre-School Children with Autism Spectrum Disorders: A cross-sectional study. *Journal of Autism and Developmental Disorders*, *53*(8), 3301–3306. <https://doi.org/10.1007/s10803-022-05498-y>

Trembath, D., Stainer, M., Caithness, T., Dissanayake, C., Eapen, V., Fordyce, K., Frewer, V., Frost, G., Hudry, K., Iacono, T., Mahler, N., Masi, A., Paynter, J., Pye, K., Quan, S., Shellshear, L., Sutherland, R., Sievers, S., Thirumanickam, A., … Tucker, M. (2023). Spoken Language Change in Children on the Autism Spectrum Receiving Community-Based Interventions. *Journal of Autism and Developmental Disorders*, *53*(6), 2232–2245. <https://doi.org/10.1007/s10803-022-05511-4>

Vicente, A., Barbarroja, N., & Castroviejo, E. (2024). Linguistic, concept and symbolic composition in adults with minimal receptive vocabulary. *Clinical Linguistics & Phonetics*, *38*(2), 155–171. <https://doi.org/10.1080/02699206.2023.2180670>

Walter, A., Martz, E., Weibel, S., & Weiner, L. (2023). Tackling emotional processing in adults with attention deficit hyperactivity disorder and attention deficit hyperactivity disorder + autism spectrum disorder using emotional and action verbal fluency tasks. *Frontiers in Psychiatry*, *14*, 1098210. <https://doi.org/10.3389/fpsyt.2023.1098210>

Wang, C.-P. (2023). Training children with autism spectrum disorder, and children in general with AI robots related to the automatic organization of sentence menus and interaction design evaluation. *Expert Systems with Applications*, *229*, 120527. <https://doi.org/10.1016/j.eswa.2023.120527>

Wang, L., Li, S., & Wang, C. (2024). Using Pivotal Response Treatment to Improve Language Functions of Autistic Children in Special Schools: A Randomized Controlled Trial. *Journal of Autism and Developmental Disorders*, *54*(6), 2081–2093. <https://doi.org/10.1007/s10803-023-05988-7>

Wang, L., Ong, J. H., Ponsot, E., Hou, Q., Jiang, C., & Liu, F. (2023). Mental representations of speech and musical pitch contours reveal a diversity of profiles in autism spectrum disorder. *Autism*, *27*(3), 629–646. <https://doi.org/10.1177/13623613221111207>

Wang, L., Xiao, S., Jiang, C., Hou, Q., Chan, A. H. D., Wong, P. C. M., & Liu, F. (2023). The form and function processing of lexical tone and intonation in tone-language-speaking children with autism spectrum disorder. *The Journal of the Acoustical Society of America*, *154*(1), 467–481. <https://doi.org/10.1121/10.0020271>

Wang, S.-H., Zhang, H.-T., Zou, Y.-Y., Cheng, S.-M., Zou, X.-B., & Chen, K.-Y. (2023). Efficacy and moderating factors of the Early Start Denver Model in Chinese toddlers with autism spectrum disorder: A longitudinal study. *World Journal of Pediatrics*, *19*(8), 741–752. <https://doi.org/10.1007/s12519-022-00555-z>

Wang, X., Delgado, J., Marchesotti, S., Kojovic, N., Sperdin, H. F., Rihs, T. A., Schaer, M., & Giraud, A.-L. (2023). Speech Reception in Young Children with Autism Is Selectively Indexed by a Neural Oscillation Coupling Anomaly. *The Journal of Neuroscience*, *43*(40), 6779–6795. <https://doi.org/10.1523/JNEUROSCI.0112-22.2023>

Weed, E., Fusaroli, R., Simmons, E., & Eigsti, I.-M. (2024). Different in Different Ways: A Network-Analysis Approach to Voice and Prosody in Autism Spectrum Disorder. *Language Learning and Development*, *20*(1), 40–57. <https://doi.org/10.1080/15475441.2023.2196528>

Wehrle, S., Cangemi, F., Janz, A., Vogeley, K., & Grice, M. (2023). Turn-timing in conversations between autistic adults: Typical short-gap transitions are preferred, but not achieved instantly. *PLOS ONE*, *18*(4), e0284029. <https://doi.org/10.1371/journal.pone.0284029>

Wehrle, S., Vogeley, K., & Grice, M. (2024). Backchannels in conversations between autistic adults are less frequent and less diverse prosodically and lexically. *Language and Cognition*, *16*(1), 108–133. <https://doi.org/10.1017/langcog.2023.21>

West, K. L., Steward, S. E., Roemer Britsch, E., & Iverson, J. M. (2024). Infant Communication Across the Transition to Walking: Developmental Cascades Among Infant Siblings of Children with Autism. *Journal of Autism and Developmental Disorders*, *54*(8), 2847–2859. <https://doi.org/10.1007/s10803-023-06030-6>

White, E. N., Cagliani, R. R., & Tyson, K. M. (2024). Effects on Speech Development With Modifications to Picture Exchange Communication System. *Focus on Autism and Other Developmental Disabilities*, *39*(1), 14–23. <https://doi.org/10.1177/10883576231178266>

Xie, F., Pascual, E., & Oakley, T. (2023). Functional echolalia in autism speech: Verbal formulae and repeated prior utterances as communicative and cognitive strategies. *Frontiers in Psychology*, *14*, 1010615. <https://doi.org/10.3389/fpsyg.2023.1010615>

Xiong, H., Liu, X., Yang, F., Yang, T., Chen, J., Chen, J., & Li, T. (2024). Developmental Language Differences in Children with Autism Spectrum Disorders and Possible Sex Difference. *Journal of Autism and Developmental Disorders*, *54*(3), 841–851. <https://doi.org/10.1007/s10803-022-05806-6>

Xu, S., Fan, J., Zhang, H., Zhang, M., Zhao, H., Jiang, X., Ding, H., & Zhang, Y. (2023). Hearing Assistive Technology Facilitates Sentence-in-Noise Recognition in Chinese Children With Autism Spectrum Disorder. *Journal of Speech, Language, and Hearing Research*, *66*(8), 2967–2987. <https://doi.org/10.1044/2023_JSLHR-22-00589>

Yankovitz, B., Kasirer, A., & Mashal, N. (2023). The Relationship between Semantic Joke and Idiom Comprehension in Adolescents with Autism Spectrum Disorder. *Brain Sciences*, *13*(6), 935. <https://doi.org/10.3390/brainsci13060935>

Yao, P.-Y., Iao, L.-S., & Wu, C.-C. (2023). Language development trajectories in young children with developmental disabilities in Taiwan. *Research in Developmental Disabilities*, *136*, 104470. <https://doi.org/10.1016/j.ridd.2023.104470>

Yerkes, B. D., Vanden Bosch Der Nederlanden, C. M., Beasley, J. F., Hannon, E. E., & Snyder, J. S. (2024). Acoustic and Semantic Processing of Auditory Scenes in Children with Autism Spectrum Disorders. *Journal of Autism and Developmental Disorders*, *54*(7), 2536–2551. <https://doi.org/10.1007/s10803-023-05924-9>

You, Y., Correas, A., White, D. R., Wagner, L. C., Jao Keehn, R. J., Rosen, B. Q., Alemu, K., Müller, R.-A., & Marinkovic, K. (2023). Mapping access to meaning in adolescents with autism: Atypical lateralization and spatiotemporal patterns as a function of language ability. *NeuroImage: Clinical*, *39*, 103467. <https://doi.org/10.1016/j.nicl.2023.103467>

Yu, W., Cheng, M., & Liang, D. (2023). The link between the factuality of verb and the theory of mind ability of Mandarin‐speaking children with high‐functioning autism. *International Journal of Language & Communication Disorders*, *58*(6), 1927–1938. <https://doi.org/10.1111/1460-6984.12910>

Zajic, M. C., McCauley, J. B., McIntyre, N. S., & Mundy, P. C. (2024). Writing Self-Concept, Text Engagement, and Writing Practices Across Contexts: Comparisons Between School-Age Children on the Autism Spectrum and Their Non-Autistic Peers. *Journal of Autism and Developmental Disorders*, *54*(10), 3792–3807. <https://doi.org/10.1007/s10803-023-06080-w>

Zane, E., & Grossman, R. B. (2023). Analysis of Noun Phrase Ambiguity in Narratives Reveals Differences in Referential Establishment But Not Cohesion for Older Autistic Children. *Journal of Speech, Language, and Hearing Research*, *66*(8), 2802–2820. <https://doi.org/10.1044/2023_JSLHR-22-00630>

Zarokanellou, V., Kotsopoulos, A., Tafiadis, D., Prentza, A., Kolaitis, G., & Papanikolaou, K. (2023). Specificity of phonological representations in school-age high-functioning ASD children. *International Journal of Speech-Language Pathology*, *25*(4), 608–618. <https://doi.org/10.1080/17549507.2022.2065030>

Zarokanellou, V., Papanikolaou, K., Tafiadis, D., & Kolaitis, G. (2023). Qualitative analysis of verbal fluency in school-age children with high-functioning autism spectrum disorders. Associations with age and IQ. *Applied Neuropsychology: Child*, *12*(3), 235–244. <https://doi.org/10.1080/21622965.2022.2090255>

Zhang, X., Song, X.-K., & So, W.-C. (2024). Examining Phenotypical Heterogeneity and its Underlying Factors in Gesture Skills of Chinese Autistic Children: Clustering Analysis. *Journal of Autism and Developmental Disorders*, *54*(9), 3504–3515. <https://doi.org/10.1007/s10803-023-06049-9>

Zhukova, M. A., Talantseva, O. I., An, I., & Grigorenko, E. L. (2023). Brief Report: Unexpected Bilingualism: A Case of a Russian Child With ASD. *Journal of Autism and Developmental Disorders*, *53*(5), 2153–2160. <https://doi.org/10.1007/s10803-021-05161-y>

Zibin, A., Altakhaineh, A. R. M., Suleiman, D., & Al Abdallat, B. (2023). The Effect of Using an Arabic Assistive Application on Improving the Ability of Children with Autism Spectrum Disorder to Comprehend and Answer Content Questions. *Journal of Psycholinguistic Research*, *52*(6), 2743–2762. <https://doi.org/10.1007/s10936-023-10019-8>
